# Reconstruction and topological characterization of the sigma factor regulatory network of *Mycobacterium tuberculosis*

**DOI:** 10.1038/ncomms11062

**Published:** 2016-03-31

**Authors:** Rinki Chauhan, Janani Ravi, Pratik Datta, Tianlong Chen, Dirk Schnappinger, Kevin E. Bassler, Gábor Balázsi, Maria Laura Gennaro

**Affiliations:** 1 Public Health Research Institute, New Jersey Medical School, Rutgers University, Newark, New Jersey 07103, USA; 2 Department of Physics, University of Houston, Houston, Texas 77204-5005, USA; 3 Texas Center for Superconductivity, University of Houston, Houston, Texas 77204-5002, USA; 4 Department of Microbiology and Immunology, Weill Cornell Medical College, New York, New York 10021, USA; 5 Max-Planck-Institut für Physik komplexer Systeme, Nöthnitzer Strasse 38, D-01187 Dresden, Germany; 6 Laufer Center for Physical & Quantitative Biology and Department of Biomedical Engineering, Stony Brook University, Stony Brook, New York 11794, USA

## Abstract

Accessory sigma factors, which reprogram RNA polymerase to transcribe specific gene sets, activate bacterial adaptive responses to noxious environments. Here we reconstruct the complete sigma factor regulatory network of the human pathogen *Mycobacterium tuberculosis* by an integrated approach. The approach combines identification of direct regulatory interactions between *M. tuberculosis* sigma factors in an *E. coli* model system, validation of selected links in *M. tuberculosis*, and extensive literature review. The resulting network comprises 41 direct interactions among all 13 sigma factors. Analysis of network topology reveals (i) a three-tiered hierarchy initiating at master regulators, (ii) high connectivity and (iii) distinct communities containing multiple sigma factors. These topological features are likely associated with multi-layer signal processing and specialized stress responses involving multiple sigma factors. Moreover, the identification of overrepresented network motifs, such as autoregulation and coregulation of sigma and anti-sigma factor pairs, provides structural information that is relevant for studies of network dynamics.

## Abstract (web summary)

Sigma factors are regulatory proteins that reprogram the bacterial RNA polymerase in response to stress conditions to transcribe certain genes, including those for other sigma factors. Here, Chauhan *et al*. describe the complete sigma factor regulatory network of the pathogen *Mycobacterium tuberculosis*.

## Graphical abstract

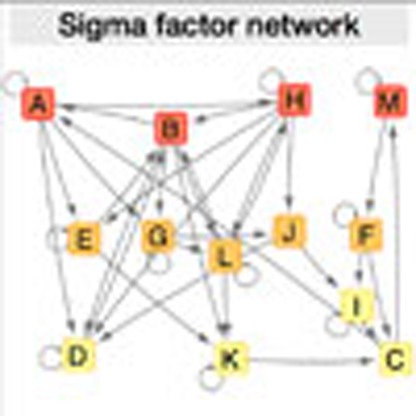

The human pathogen *Mycobacterium tuberculosis* (*M. tuberculosis*) causes millions of cases of tuberculosis each year^1^. The infected host generates an immune response that the bacteria counter by extensive transcriptional and metabolic remodelling. The bacterial response ultimately leads to bacterial growth arrest and reduced susceptibility to host defence mechanisms. A chronic, asymptomatic condition ensues (latent *M. tuberculosis* infection). When host defenses weaken in latently infected individuals, tubercle bacilli resume growth and pulmonary disease develops. Infection can then be transmitted from diseased to uninfected individuals. Understanding how *M. tuberculosis* responds and adapts to host-generated stress is crucial for developing effective anti-tuberculosis strategies.

In bacteria, responses to stress involve remodelling of cellular programs at both the transcriptional and translational levels^2^,^3^. Implementing stress responses requires sensing and processing information that arrives from the internal and external environment in the form of biochemical and physical changes^4^. Bacteria have evolved multiple stress responses that include two-component systems, protein-modifying and -degrading enzymes, molecular chaperones and accessory sigma factors^5^,^6^,^7^,^8^. Expression of accessory sigma factors, which are found in all bacteria examined except *Mycoplasma*^8^, leads to the reprogramming of RNA polymerase (RNAP) by a change in the sigma factor, the subunit that ensures specificity of the RNAP holoenzyme for specific promoter sequences and, consequently, initiation of transcription of particular gene sets^8^. The ‘housekeeping' sigma factor typically directs RNAP to genes needed for essential functions in normal growth conditions, while accessory sigma factors reprogram RNAP to transcribe genes involved in stress responses. The tubercle bacillus has 13 sigma factors (1 housekeeping and 12 accessory)^9^,^10^, suggesting that *M. tuberculosis* can respond to diverse, complex stimuli.

Transcription of each sigma factor gene requires an RNAP associated with a sigma factor. Therefore, sigma factors regulate each other, forming a sigma factor network that is critical for the pathogen's stress response and survival. Interactions among sigma factors help reveal the logic of stress responses. For example, when one type of stress typically precedes another, the sigma factor(s) associated with the first stress may regulate the sigma factor(s) associated with the second. Such is the case of the sigma factor cascade that regulates sporulation in *Bacillus subtilis* (*B. subtilis*)^11^: sequentially expressed sigma factors represent progressive cellular commitment to spore formation and presumably imply increasing levels of stress. If multiple stress conditions tend to co-occur, the relevant sigma factors may regulate each other^12^. In contrast, sigma factors that do not cross-talk may enable insulated expression of the corresponding regulons under particular stress conditions that do not tend to co-occur^13^. Despite its biological importance, the regulatory connectivity among sigma factors has not been systematically investigated in *M. tuberculosis*.

In the present work, we integrate identification of direct regulatory interactions between all *M. tuberculosis* sigma factor pairs in a synthetic *Escherichia coli* (*E. coli*) expression system, validation of selected links in *M. tuberculosis*, and extensive review of the literature to obtain the first complete sigma factor regulatory network of *M. tuberculosis*. Network analysis indicates that the network partitions into clearly separable network communities. The network displays hierarchical organization, high internal connectivity and extensive autoregulation. Furthermore, embedding the sigma factor network into the known transcription-regulatory network of *M. tuberculosis* reveals a tendency for cognate sigma and anti-sigma factors to be coregulated.

## Results

### An *E. coli* two-plasmid assay for sigma–sigma interactions

**Figure 1:**
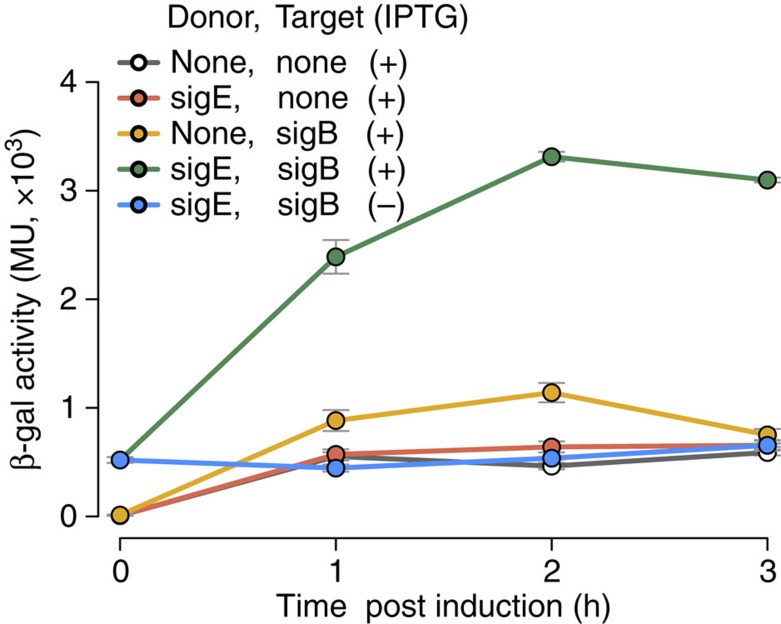
***E. coli*** **two-plasmid system testing the known direct** ***sigE*****–*****sigB*** **interaction of** ***M. tuberculosis***. An *E. coli* BL21 (DE3) strain was constructed containing a donor plasmid expressing *sigE* under an IPTG-inducible promoter and a target plasmid carrying a *sigB::lacZ* reporter fusion. Control strains contained either plasmid with the corresponding empty partner plasmid, or both empty plasmids. Mid-log phase cultures were treated with 100 μM IPTG, and collected before treatment (time 0) and at hourly intervals post treatment. β-galactosidase assays were performed with aliquots of cell lysates using o-nitrophenyl-β-D-galactopyranoside as substrate. Miller units (MU) were calculated as in Methods. Data are presented as mean values (±s.d.) from triplicate experiments. Each colour represents a different strain. pACYCDuet-1, empty donor vector; pJEM13, empty target vector; pACYC::*sigE*, donor vector expressing IPTG-inducible *sigE*; pJEM13::*sigB*, target vector carrying a *sigB::lacZ* fusion.

We set out to identify direct regulatory interactions among accessory sigma factors of *M. tuberculosis* using an *E. coli* two-plasmid system. In one plasmid set, each *M. tuberculosis* sigma factor gene was expressed under an isopropyl beta-D-1-thiogalactopyranoside (IPTG)-inducible promoter (donor). The second set of plasmids expressed reporter *lacZ* fused to the promoter of each of the *M. tuberculosis* sigma factor genes (target). *E. coli* strains containing all combinations of donor–target pairs were tested for β-galactosidase activity in IPTG-treated cultures to determine the ability of each donor sigma factor to induce expression of each target sigma factor promoter. The *E. coli* system was selected for two reasons. First, the use of core *E. coli* RNA polymerase (RNAP) to test *M. tuberculosis* sigma factor activity in transcription assays *in vitro* (for example, ref. 14) indicates that *M. tuberculosis* sigma factors can utilize *E. coli* core RNAP. Second, the interactions between two *M. tuberculosis* sigma factors in a heterologous system, such as *E. coli*, are expected to be direct rather than indirect, because *E. coli* is unlikely to encode putative ‘intermediate' factors connecting the sigma factor pair being tested. We assessed the two-plasmid *E. coli* assay in a proof-of-principle experiment involving ‘donor' *sigE* and ‘target' *sigB::lacZ*, since *sigB* carries a σ^E^-dependent promoter^15^. Induction of *sigE* with IPTG resulted in increased β-galactosidase activity (Fig. 1), demonstrating that the assay functioned.

### *E. coli* assays using a 13 × 13 sigma factor matrix

**Figure 2:**
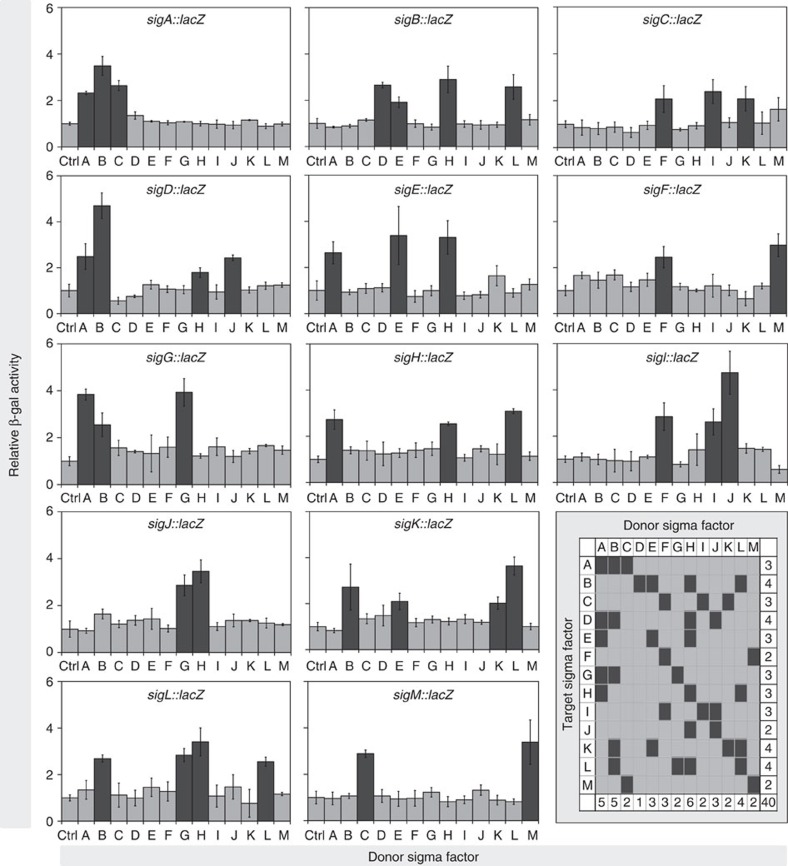
**β-galactosidase assay results for the 13 × 13 matrix of** ***M. tuberculosis*** **sigma factors.** *E. coli* strains containing all pairs in the 13 × 13 matrix were used for β-galactosidase assays performed using the medium-throughput protocol (Methods). Each panel shows results for a single target *sig::lacZ* fusion tested against each of 13 sigma factor donor plasmids, plus the empty donor plasmid (control plasmid, Ctrl). Data were expressed as Miller units (MU), as described in Methods. Relative β-galactosidase activity was calculated as 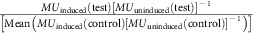. Data are presented as mean values (±s.d.) from triplicate experiments. Black bars represent significant interactions (*P*<0.05, tested by ANOVA). The last panel represents a summary of the interaction data between target sigma factors (rows) and donor sigma factors (columns). Black boxes at the intersection between rows and columns represent interactions of the corresponding sigma pair. Each black box matches a black bar in the panel of the corresponding target sigma factor. The last column and row in the margins of the summary matrix represent the total number of interactions in the corresponding row (target sigma) and column (donor sigma), for a grand total of 40 (bottom right-corner box).

The *M. tuberculosis* genome encodes 1 essential sigma factor (*sigA*) and 12 accessory sigma factors (*sigB* through *sigM*)^9^,^10^. We constructed *E. coli* strains carrying donor plasmids for each of 13 sigma factors. Induction of gene expression with IPTG was not toxic for *E. coli* (Supplementary Fig. 1), and recombinant proteins were detected on IPTG induction by western blot analysis (Supplementary Fig. 2). We also constructed *E. coli* strains carrying target plasmids for each sigma factor of *M. tuberculosis.* Subsequently, *E. coli* strains containing all plasmid pairs (13 donors by 13 targets) were generated and used to assay β-galactosidase activity. Results of these assays are shown in Fig. 2.

When we assessed significance for each donor–target pair, we obtained a total of 40 significant interactions, including autoregulation (*P*<0.05 by analysis of variance and *post hoc* tests; summary panel in Fig. 2). We compared our data with the direct sigma–sigma interactions previously reported with *in vitro* transcription assays conducted by various laboratories and with two chromatin immunoprecipitation (ChIP)-based studies (ChIP-on-chip^16^ and ChIP-seq^17^). Of the 15 direct links reported previously (Supplementary Table 1), 11 (73%) were revealed by our assay.

**Figure 3:**
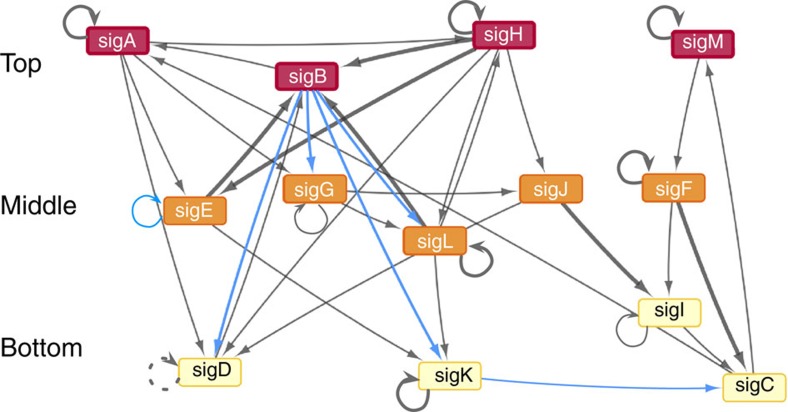
**The sigma factor regulatory network of** ***M. tuberculosis***. The interactions identified in the 13 × 13 matrix assay were plotted as a directed network, with the nodes representing sigma factors, and the edges representing direct regulatory interactions. Thick lines represent interactions reported in the literature before the present work (Supplementary Table 1): the autoregulation of *sigD*, marked with a dashed line, is the only high-confidence link previously detected by multiple assays that was not detected with the *E. coli* 13 × 13 assay. Thin lines represent novel interactions: in blue are those tested and validated in *M. tuberculosis* in the present work. The node colours represent three levels of network hierarchical organization, which was estimated using a probabilistic approach (Supplementary Fig. 3).

We also analysed the four interactions described in the literature but not revealed by our assay. We found that (1) the autoregulation of *sigD* had been observed with transcription assays *in vitro*^18^ and with both ChIP studies; (2) the autoregulation of *sigB* was reported in one *in vitro* transcription study^14^ but not in another^19^, and it was not seen in ChIP studies; (3) the regulation of *sigB* by SigF was observed by *in vitro* transcription^19^ but not by ChIP studies; and (4) the regulation of *sigE* by SigL was only detected by ChIP-seq (this result might be an artefact of overexpression, since similarities exist between SigL, SigE and SigH binding sites^20^,^21^ and since SigH and SigE bind upstream of *sigE* (refs 17, 22 and Fig. 2)). Based on the above considerations, we added the *sigD* autoregulatory link to our network reconstruction. We considered the three remaining links to be of lower confidence, given disagreements among previous reports; we did not add them to the reconstructed network. With the results of the 13 × 13 *E. coli* assay plus *sigD* autoregulation, we obtained a sigma factor regulatory network of 41 direct interactions among 13 sigma factors (Fig. 3).

### Validation of sigma factor interactions in *M. tuberculosis*

**Figure 4:**
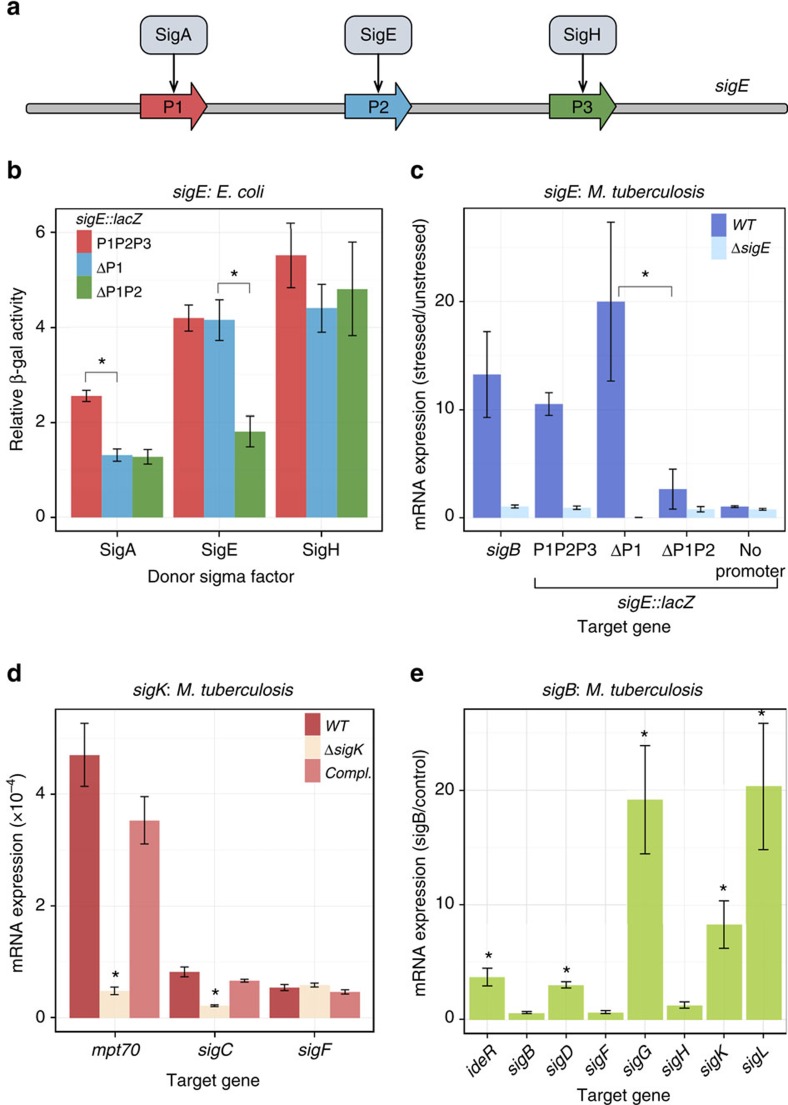
**Experimental validation of selected links in** ***M. tuberculosis***. (**a**) Schematic representation of the *sigE* promoter region. The three promoters upstream of *sigE*^23^ are recognized by SigA, SigE and SigH, respectively (this work and ref. 22). A search for consensus binding sites for SigA, SigE and SigH in the nucleotide sequences present in each promoter::*lacZ* fusion found no matches for SigA and SigE; instead, a match for SigH was found at the appropriate location upstream of the P3 transcription start site. (**b**) Effects of *sigE* promoter deletions on reporter β-galactosidase activity in *E. coli*. *sigE::lacZ* target plasmid and promoter-deletion derivatives were tested in cells containing sigma donor plasmids or a control plasmid (empty vector), as indicated. Relative beta-galactosidase activity was calculated as in the Fig. 2 legend. In this and the next panel, P1P2P3, native promoter configuration; ΔP1, deletion of P1; ΔP1P2, deletion of P1 and P2. (**c**) Effects of *sigE* promoter deletions on *sigE::lacZ* expression in *M. tuberculosis*. Mid-log-phase cultures of wild type and *sigE* deletion mutant of *M. tuberculosis* H_37_Rv containing a *sigE::lacZ*-carrying plasmid and promoter-deletion derivatives were treated with 0.03% SDS for 60 min. In this and subsequent panels, cells were collected, RNA isolated, and transcripts enumerated and normalized to 16S rRNA. mRNA levels were normalized relative to unstressed controls. No promoter=empty vector control. (**d**) Effect of *sigK* deletion on selected gene expression in *M. tuberculosis*. Mid-log-phase cultures of *M. tuberculosis* H_37_Rv, a *sigK* deletion mutant, and a complemented strain were used for transcript enumeration. (**e**) Effect of *sigB* induction on selected gene expression in *M. tuberculosis*. Mid-log-phase cultures of *M. tuberculosis* H_37_Rv containing an anhydrotetracycline (ATC)-inducible copy of s*igB* or a control (empty vector) construct were treated with 1.6 μg ml^−1^ ATC for 24 h and used for transcript enumeration. mRNA levels were normalized to empty-vector control. Data in panels **b**–**e** are presented as mean values (±s.e.m.) from triplicate experiments. Asterisk marks denote significance of the comparisons indicated (*P*<0.05 in one-sided *t*-test, and fold change >1.5).

To validate the sigma–sigma interactions depicted in Fig. 3, we first attempted use of published consensus sigma factor binding sequences to predict donor sigma factor binding upstream of target sigma factor genes (see Methods). The analysis identified only 6 out of 15 (<50%) previously reported direct sigma–sigma interactions (Supplementary Table 2), indicating poor predictive power of these consensus sequences. We thus turned to assays in *M. tuberculosis* to test some of the novel interactions detected in the *E. coli* 13 × 13 matrix assay. First, we investigated the results obtained for *sigE* in the *E. coli* assay, in which the *sigE* promoter region was recognized by three donor sigma factors, SigA, SigE and SigH. Earlier work showed that *sigE* can be transcribed from three promoters, P1, P2 and P3 (ref. 23). To analyse the relationship between each of the three donor sigma factors and each *sigE* promoter, we introduced progressive deletions into the promoter region of the *sigE::lacZ* promoter fusion and tested the deletion products for β-galactosidase activity in *E. coli* in the presence of each of the three sigma donors. We found that the effects of donor SigA and SigE required the presence of P1 and P2 DNA, respectively, on the target *sigE::lacZ* construct. Moreover, donor SigH was responsible for gene induction at P3 (Fig. 4a,b). These results led to several conclusions. First, transcription from promoter P1 involves the housekeeping sigma factor SigA. This is consistent with the lack of P1 regulation in response to known stress conditions except surface stress (during surface stress P1 is bound by and downregulated via steric hindrance by the transcription factor MprA, which is required for the surface stress response of P2 (ref. 23)). Second, our results agree with P3 being a SigH-dependent promoter^22^. Third, our *E. coli* data show that *M. tuberculosis* SigE-containing RNAP interacts with DNA in the P2 region and transcribes *sigE*.

Tests with *M. tuberculosis* using a series of *sigE::lacZ* constructs bearing progressively shorter upstream regulatory sequences revealed that the surface-stress response of the reporter transcript, which is P2 dependent^23^, was abrogated either by deletion of P2 from the construct in wild-type cells or by genetic inactivation of *sigE* in the bacterial chromosome (Fig. 4c). These results are consistent with functional SigE being required for stress-responsive transcription at P2. However, since *sigE* and *mprAB* regulate each other's transcription^24^ and since *mprAB* is required for the P2 surface stress response^23^, the genetic analysis in *M. tuberculosis* did not distinguish between direct and indirect effects of *sigE* on the P2 stress response. Overall, these results show that all three sigma factors are involved in the transcription of the key stress-responsive *sigE* of *M. tuberculosis*^25^, and they support a hitherto unrecognized role for the P2 promoter in *sigE* autoregulation.

A second, new interaction revealed in the 13 × 13 matrix assay is *sigC* targeting by SigK. We found that, during exponential growth, expression of *sigC* was reduced almost four-fold in an *M. tuberculosis sigK* deletion mutant, and that complementation fully restored *sigC* expression (Fig. 4d). Similar effects of the *sigK* mutation and mutant complementation were obtained with the known SigK target *mpt70*, which served as a positive control^26^, while no effect was seen with a negative, non-target control (*sigF*) (Fig. 4d). Thus, the *M. tuberculosis* data validate the *sigK–sigC* interaction observed in the *E. coli* test system.

Third, using an *M. tuberculosis* strain containing a copy of *sigB* controlled by an anydrotetracycline (ATC)-inducible promoter, we examined the interactions seen in the *E.* coli-based assay between *sigB* and four target sigma factors: *sigD*, *sigG*, *sigK* and *sigL*. We also tested the potential autoregulation of *sigB* reported in some *in vitro* transcription assays^14^ but not in others^19^, which we had not detected in the *E. coli* test system. Treatment with ATC induced all four sigma factor genes that were identified as SigB targets in the 13 × 12 matrix assay and the positive control *ideR*, a known SigB target^14^ (Fig. 4e). No induction was seen with the native copy of *sigB* or with two negative, non-target controls (*sigH* and *sigF*). Thus, we confirmed with *M. tuberculosis* the *sigB* results obtained in the *E. coli* assay, including the absence of *sigB* feedback regulation.

In conclusion, we validated each of the six new interactions by tests with *M. tuberculosis* (blue lines in Fig. 3). This result strongly suggests that most of the 23 links in the network that remain untested will also be *bona fide*, direct regulatory interactions.

### Network hierarchy

Cellular regulatory networks often exhibit a hierarchical organization in which some nodes function as top-level master regulators while others act downstream as effectors^27^. In the case of sigma factors, one might envision that the farther downstream a sigma factor is, the more specific the stress signal it responds to. In contrast, sigma factors in the top layers of the hierarchy might respond to multiple stresses or even participate in the general stress response that reduces damage until more specific stress responses are expressed to eliminate it^28^,^29^. We assessed the hierarchy in the *M. tuberculosis* sigma factor network by applying a hierarchy score maximization algorithm^30^, which is based on probabilistic assignment of nodes to hierarchical levels to achieve maximal downward flow. When we used the corrected hierarchy score and a node-ambiguity score, we found that a three-level hierarchy best describes the *M. tuberculosis* sigma factor regulatory network (Supplementary Fig. 3a). Then we used probabilistic assignment of nodes to place each sigma factor in one of the three hierarchical levels. The results were as follows: (i) top level: *sigA, sigB*, *sigH*, *sigM*; (ii) middle level: *sigE*, *sigF*, *sigG*, *sigJ*, *sigL*; and (iii) bottom level: *sigC*, *sigD*, *sigI*, *sigK* (Supplementary Fig. 3b). The network in Fig. 3 reflects this hierarchical organization. We obtained similar assignment of sigma factors to hierarchical layers from the in- and out-degrees of connectivity when we analysed individual nodes^31^ rather than overall network properties. These results indicate that the sigma factor regulatory network of *M. tuberculosis* has a hierarchical structure, with master regulators *sigA*, *sigB*, *sigH* and *sigM* feeding signals into the network that are then processed by the remaining sigma factors.

### Community structure

**Figure 5:**
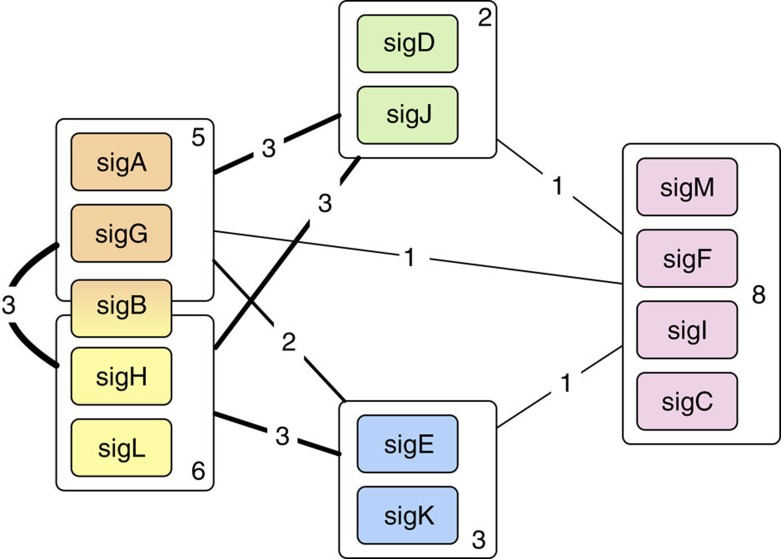
Communities within the sigma factor regulatory network. The figure shows the five communities identified in the sigma factor network. Each community is represented by a box containing the sigma factors that are members of that community. The lines represent connections between two communities. Line thickness and the numbers on the lines indicate the number of links between two communities, regardless of directionality. The number inside each box represents the number of links among sigma factors within each community. The membership of *sigB* and corresponding protein in two different communities is shown by its position and box colour.

We next asked whether groups of sigma factors exist that are preferentially connected to each other rather than to other sigma factors, thereby forming communities^32^. The existence of communities might identify sigma factors responding together to one or more particular stress signals. To address this possibility, we converted the directed network shown in Fig. 3 into a bipartite network in which each sigma factor has a gene node and a protein node. We then used a bipartite modularity algorithm to identify ‘biclustered' communities consisting of both gene and protein nodes (see Methods). By applying the algorithm 10,000 times, we found a stable partition comprising five sigma factor communities. The results were expressed as a ‘heat-map' correlation matrix for the probability that each pair of nodes is in the same community (Supplementary Fig. 4). By comparing the community structure of the sigma factor bipartite network with an ensemble (size=10,000) of random bipartite networks having the same number of nodes and links, we consistently found that the modularity effect-size of the community structure is highly significant (*z*-score=96.24) (Supplementary Fig. 4). The largest core community included the *sigC*, *sigF*, *sigI* and *sigM* genes and the corresponding proteins. Two additional core communities were (i) *sigA* and *sigG* (and corresponding proteins) plus the SigB protein, and (ii) *sigH* and *sigL* (and corresponding proteins) plus the *sigB* gene. Finding the *sigB* gene and SigB protein in two different communities suggests that SigB may serve as a bridge between these two communities. The remaining two small communities linked *sigE* to *sigK* and *sigJ* to *sigD*. Enumerating the links within and among communities showed that the tightly knit *sigC*, *sigF*, *sigI* and *sigM* community is the most segregated from the rest of the network (Fig. 5). Indeed, several other community detection algorithms (including refs 33, 34, 35) consistently assigned *sigC*, *sigF*, *sigI* and *sigM* to the same community. Moreover, the four sigma factors in this community, together with *sigJ* and *sigK* that bridge it to the rest of the network (Fig. 3), tend to be coexpressed across multiple conditions (Supplementary Fig. 5). Thus, community analysis provides a robust indication for the existence of a small, distinct island of four sigma factors that may coordinately respond to the same environmental stimuli.

### Other topological network properties

We next examined key regulatory and topological properties of the sigma factor regulatory network and compared them with the *M. tuberculosis* regulatory network of transcription factors devoid of sigma factors to determine whether the sigma factor network exhibits distinctive properties. We first analysed autoregulation, a topological feature that can affect network dynamics by modulating response times^36^. Of the 41 links in the sigma factor regulatory network, 10 are autoregulatory loops (Fig. 3). Thus, the probability of autoregulation is 10/41=0.24, which is three-fold greater than the value 13/169=1/13=0.077 expected by chance. Indeed, link randomization resulted in networks with significantly fewer autoregulated sigma factors (*n*=3.15±1.5) than the 10 autoregulatory links observed in the actual sigma factor network (*P*<10^−4^). Moreover, we calculated similar frequencies of autoregulation within sub-networks of randomly selected *M. tuberculosis* transcription factors (generated using ChIP-seq data^17^). Indeed, occurrence of autoregulation more frequently than expected by chance has also been observed in transcriptional networks of other microorganisms, such as *E. coli*^37^. Thus, autoregulation is not a distinguishing feature of the *M. tuberculosis* sigma factor network even though it is more prevalent than expected by chance.

**Figure 6:**
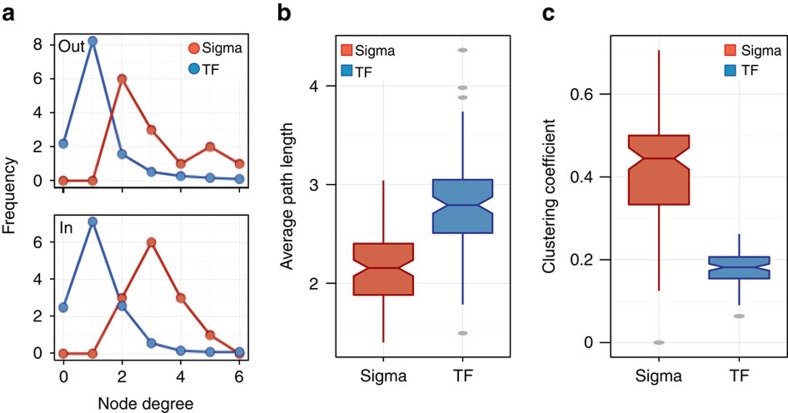
Topological properties of the sigma factor and transcription factor networks. The figure shows comparisons of network properties between the sigma factor network (red) and randomly selected sub-networks from the transcription factor (TF) network (blue) of *M. tuberculosis* (which includes 67 transcription factors other than sigma factors^17^). (**a**) Out- and in-degree distributions of the sigma factor network and transcription factor sub-networks of comparable size (average of 100 samples of 13-node sub-networks derived from the TF network). (**b**) Average path length and (**c**) clustering coefficient distributions for the sigma factor and transcription factor networks were calculated based on resampling two-thirds of the total nodes in the respective networks (for both **b** and **c** *P* values were <<0.01 by Wilcoxon's rank-sum test).

Graph-theoretical properties, such as degree distribution, clustering coefficient and average path length, provide quantitative insight into the architecture of a network. We first calculated the in- and out-degree distribution profiles of the sigma factor network (Fig. 6a). We then compared these profiles with the median degree distribution of sub-networks randomly selected from the transcription factor network. To perform this comparison, we randomly sampled transcription factor sub-networks having the same number of nodes as the sigma factor network (*n*=13) and calculated the median distribution obtained from the sampled sub-networks. We observed a rightward shift towards higher in- and out-degrees for the sigma factor network relative to the randomly selected transcription factor sub-networks (Fig. 6a). In addition, when we sampled random sub-networks from the sigma factor network and the transcription factor network, we found significantly lower average path length and higher clustering coefficient for the sigma factor network (Fig. 6b,c; *P*<2E-16). These differences may result from the higher node degree of the sigma factor network. Together, these three network topological measures indicated that the sigma factor regulatory network is more interconnected than the regulatory network of transcription factors in the same microorganism.

We next reasoned that nodes located at different levels of the hierarchy of the sigma factor network might have different impact on network connectivity. To test this possibility, we analysed the effect of *in silico* deletion of each sigma factor on the clustering coefficients of the resulting network. Deleting top-level nodes resulted in networks having lower clustering coefficients relative to the wild-type network, while the opposite effect was observed when bottom-level nodes were deleted (Supplementary Fig. 3c). Thus, as expected, top-level nodes are most critical to the connectivity of the sigma factor network, further supporting the hierarchical network organization displayed in Fig. 3.

### Sigma factor and transcription-regulatory networks

**Figure 7:**
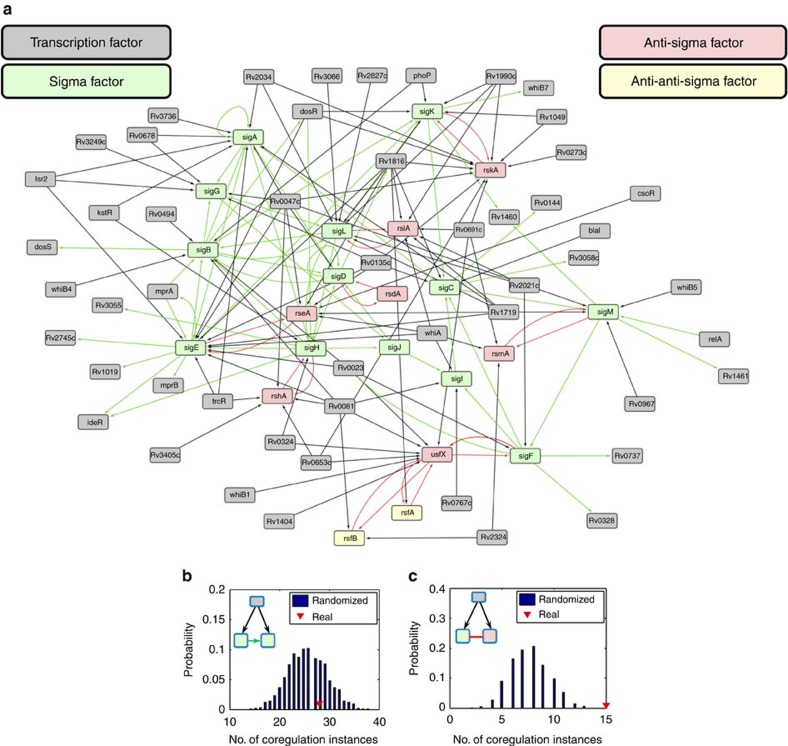
**Embedding the sigma factor regulatory network into the transcription-regulatory network of** ***M. tuberculosis***. (**a**) Transcription-regulatory neighbourhood of the sigma factor network. Each rectangle represents a gene and its protein product. Colours: grey, transcription factors; green, sigma factors; red, anti-sigma factors; yellow, anti-anti-sigma factors. Arrowheads indicate the direction of the regulatory links. (**b**) Number of occurrences for the network structure (inset) where a transcription factor regulates two sigma factors, one of which also regulates the other. Red triangle: actual number of occurrences; blue bars: distribution of occurrences in randomized networks. (**c**) Number of occurrences for the network structure (inset) where a transcription factor regulates a sigma factor and its corresponding anti-sigma factor. Red triangle and blue bars are as in panel (**b**).

Since sigma factors are required for the transcription of transcription factors and since transcription factors can regulate sigma factor expression (a well-known example is the mutual regulation between *sigE* and the two-component system *mprAB*^23^), we asked how the sigma factor network integrates into the larger transcription-regulatory network of *M. tuberculosis*. For this analysis, together with sigma factors we included the known anti-sigma and anti-anti-sigma factors (Supplementary Table 3), which regulate (and may be regulated by) the levels of active sigma factor in the cell^38^,^39^. To understand how the sigma factor network is embedded into the larger transcription-regulatory network, we mapped the immediate network neighbourhood, which includes transcription factors that directly regulate a sigma factor, an anti-sigma factor or an anti-anti-sigma factor, and transcription factors for which sigma factor(s) binding to their promoter has been characterized. The resulting mixed network is shown in Fig. 7a.

We observed that certain transcription factors are sigma-specific (for example, *relA* only regulates *sigM* and Rv1648c only regulates *sigC*). Others tend to regulate multiple sigma factors and anti-sigma factors (for example, Rv0691c regulates four anti-sigma factors and *mprA* regulates two sigma factors), presumably coordinating combinatorial responses to various stress conditions. We also observed that certain transcription factors regulate a sigma factor and its cognate anti-sigma factor, forming feed-forward loop structures (for example, Rv1049 and Rv1990c regulate both *sigK* and its cognate anti-sigma factor gene *rskA*). To assess the representation of such network motifs, we compared their number in the *M. tuberculosis* network with that obtained from randomized networks. We found no overrepresentation for regulators controlling two sigma factors that regulate each other (Fig. 7b). Likewise, two sigma factors regulating each other do not tend to control the same transcription factor. Instead, we observed an unexpectedly large number of cases in which a transcription factor regulates both a sigma factor and the cognate anti-sigma factor (Fig. 7c and Supplementary Fig. 6). This excessive coregulation is not explained solely by the presence of sigma and anti-sigma factor genes in the same operon, which is frequently observed (Supplementary Table 3 and references therein). Rather, we find that transcription factors tend to coregulate sigma and anti-sigma factor pairs even when the two genes are transcribed from different promoters (one known example is *sigE* and *rseA*, see Supplementary Fig. 6). These results point to the presence of regulatory network motifs that result in coexpression of sigma factors and their cognate anti-sigma factors in *M. tuberculosis*.

## Discussion

In the present study, we report that a simple approach utilizing a tractable model organism, such as *E. coli*, made it possible to reconstruct the full network of direct transcriptional interactions among all 13 sigma factors of the human pathogen *M. tuberculosis*. Network analysis identified three main topological features of the network. First, the sigma factor network is densely connected, implying that multiple direct and indirect pathways exist between most sigma factor pairs. Second, the network has three hierarchical levels, with master regulators located at the top of the hierarchy. Third, the network contains a tight community of four sigma factors that is clearly separable from the rest of the network. These network features collectively suggest the ability (i) to implement initial generic stress responses that ensure survival before expression of stress-specific responses by the deeper parts of the network (hierarchical organization), (ii) to implement combinatorial or redundant responses to diverse (or complex) stress conditions (high connectivity) and (iii) to engage multiple sigma factors in specific stress responses (community structure). Moreover, our study identified overrepresented regulatory motifs in the network that are expected to have functional implications. One is coregulation of sigma and anti-sigma factors, which likely leads to rapid sigma factor deactivation when stress stimuli stabilize or dissipate^40^. The other is autoregulation. The effect of autoregulation on network dynamics depends on its sign (positive or negative)^36^. Since sigma factors promote transcription, sigma factor autoregulation *per se* is positive and might therefore lead to delayed response time^36^,^41^, ensuring that the cell invests in stress responses only when the external stress is prolonged. However, when the sigma factor regulates the cognate anti-sigma factor, the net sign of the feedback regulation depends on biochemical properties that cannot be predicted by the regulatory network structure. A complete understanding of network dynamics will require full integration of the transcriptional network structure provided by the present work with small-scale analysis of the regulatory feedbacks resulting from the complex post-transcriptional regulation of sigma factor activity by anti-sigma and anti-anti-sigma factors^38^,^39^.

How do sigma factor communities in the network correlate with stress responses? One known correlation is between stationary growth phase and the most distinct community in the network, that composed of *sigC*, *sigF*, *sigI* and *sigM* (Fig. 5). This community is likely governed by *sigM* as local master regulator and is linked to the rest of the network through *sigK* and *sigJ* (Fig. 3). Expression of *sigM* is induced in stationary phase in *M. smegmatis*, *M. bovis* BCG and *M. tuberculosis*^42^,^43^. Similarly, *sigJ*, which regulates *sigI* (Fig. 3; also ref. 44 and ChIP-seq data^17^), is expressed at high levels in late stationary-phase cultures of *M. tuberculosis*. Likewise, *sigF* is strongly induced during stationary phase, at least in *M. bovis BCG*^45^. Although no similar induction has been observed in *M. tuberculosis*^46^, genetic inactivation of *sigF* in this pathogen results in reduced expression of genes predominantly in stationary-phase cultures^47^. Among the *sigF*-regulated genes is *sigC* (ref. 14 and Fig. 3), which is also regulated by *sigK* (Figs 3 and 4d). Moreover, *sigD*, which is associated with *sigJ*, has also been connected with stationary phase^48^,^49^. Furthermore, members of the subset (*sigM*, *sigI*, *sigK* and *sigJ*) exhibit decreased expression in response to culture medium supplementation with fatty acids (expression data in www.tbdb.org), and *sigF* is induced by nutrient starvation^50^. These observations support a role for this community in the response to nutrient limitation, as it occurs in the stationary growth phase of axenic cultures. In addition, sigma factors in this community tend to be coexpressed across various test conditions, based on analysis of published gene expression data sets (Supplementary Fig. 5). Thus, correlations between community structure and specific stress responses exist for the most clearly defined community in the network.

The results of the *E. coli*-based approach significantly expand current knowledge on the sigma factor network of *M. tuberculosis*. Other methods for revealing direct interactions among sigma factors have limitations. First, *in vitro* transcription measurements, which are cumbersome, do not lend themselves to genome-wide analyses. Second, ChIP-based work (initially ChIP-on-chip, and then much broader ChIP-seq studies^16^,^17^,^51^) analysed genome-wide DNA binding for 11 of 13 sigma factors (see also http://networks.systemsbiology.net/mtb/). Yet this work revealed only seven significant sigma–sigma interactions (summarized in Supplementary Table 1) and did not identify significant consensus sequences for any of the sigma factors tested^17^. These results suggest that current ChIP methodologies may not be ideal when applied to sigma factor binding, which occurs only when RNAP core and sigma factor form the holoenzyme^8^. Moreover, when comparing data across the above techniques, it is worth noting that the *E. coli*-based approach and *in vitro* transcription measurements characterize gene transcription, while ChIP-based methodologies reveal RNAP binding to DNA, which may or may not give rise to RNA synthesis^52^. Thus, results obtained with these various techniques need to be viewed as complementary. Third, analysis of gene induction following regulated sigma factor overexpression cannot identify direct interactions *per se*. Nonetheless, it can corroborate direct-interaction analyses, such as our *E. coli* approach (for example, Fig. 4e), *in vitro* transcription, or ChIP methods (see examples in Supplementary Table 1). Other, more indirect methods, such as those utilizing bacterial mutants, pose even greater hurdles to data interpretation. For example, genetic inactivation of a sigma factor gene may or may not result in reduced expression of direct, downstream target genes (for example, ref. 53), possibly due to potential regulatory redundancies. In light of the above considerations, we conclude that, given its genome-wide scope, our approach fills a considerable knowledge gap in *M. tuberculosis* sigma factor biology.

Reconstruction of the sigma factor network of *M. tuberculosis* opens multiple avenues of future research. One is the study of network dynamics. As mentioned above, the functional implications of the overrepresentation of particular network motifs require integrating transcriptional and post-transcriptional mechanisms, given the complex regulation of sigma factor activity. A second research avenue is integrating the sigma factor regulatory network with the transcription factor regulatory network since transcriptional responses result from the integrated activity of these two classes of regulators. Additional data on sigma factor binding to the promoters of transcription factors will be required to further link the two networks. A third area of research involves using network structure information to study sigma factors and stress responses across bacterial species. For example, work in model organisms, such as *E. coli* and *B. subtilis*, has revealed hierarchical and modular organization of the transcription-regulatory network (including sigma factors)^54^,^55^. The network structure characterized in the present work should facilitate in-depth comparative studies that identify functional orthologues across species (for example, it is currently possible to identify clusters of orthologous groups between *M. tuberculosis* and *B. subtilis*, but not to establish one-to-one correlations between individual sigma factors of these two microorganisms). Such comparative studies should facilitate further understanding of connections between individual sigma factors, sigma factor communities, and stress response functions. This knowledge might in turn generate a mechanistic insight of environment sensing, signal processing and survival to stress by *M. tuberculosis*, and ultimately lead to finding potential targets for novel antibiotics.

## Methods

### Bacterial strains and reagents

*E. coli* XL-1 blue (Agilent Technologies, Santa Clara, CA) was used for DNA cloning procedures, while *E. coli* BL21 (DE3) (EMD Biosciences, Madison, WI) was used for expression of *M. tuberculosis* sigma factors. Plasmid pACYCDuet-1 (EMD Biosciences, Madison, WI) was used to clone and express *M. tuberculosis* sigma factor genes as S-tagged proteins under an IPTG-inducible T7 promoter. The promoter-probe plasmid pJEM13 (ref. 56) was used to construct fusions of sigma factor promoters with the *lacZ* reporter gene. *E. coli* cultures were propagated at 37 °C in Luria–Bertani medium (LB). *E. coli* transformants were selected on LB-agar plates containing kanamycin (40 μg ml^−1^), chloramphenicol (25 μg ml^−1^), or both, as required. Blue–white selection of transformants was performed with X-gal (20 μg ml^−1^) (Invitrogen, Carlsbad, CA). Cultures of *M. tuberculosis* H_37_Rv (ATCC 27294) were grown in Difco Middlebrook 7H9 (liquid medium) or on 7H10 (solid medium) supplemented with 0.05% Tween 80 (Sigma-Aldrich, St Louis, MO), 0.2% glycerol (Sigma-Aldrich), and 10% ADN (2% dextrose, 0.5% BSA and 0.15 M NaCl). Liquid cultures of *M. tuberculosis* were grown in tubes at 37 °C with magnetic-bar stirring at 450 r.p.m. Plates were incubated at 37 °C in sealed plastic bags. *M. tuberculosis* transformants were selected on 7H10 agar plates supplemented with kanamycin (20 μg ml^−1^) or hygromycin (50 μg ml^−1^), as needed.

### Sigma factor expression and reporter plasmid construction

Sigma factor genes were amplified from genomic DNA of *M. tuberculosis* H_37_Rv by PCR using forward and reverse primers (Supplementary Table 4). Amplification was carried out using Taq DNA polymerase (Roche Applied Science, Indianapolis, IN). Amplified DNA was digested with appropriate restriction endonucleases and cloned in the multiple cloning site 2 of pACYCDuet-1 to create a fusion with the plasmid-borne S-tag at the C-terminal end of the recombinant product. Recombinant colonies were selected on LB-agar plates supplemented with chloramphenicol.

A ∼500-bp fragment containing upstream sequences and the initial 45 bp of the predicted open read frame was amplified by PCR from the chromosomal DNA of *M. tuberculosis* H_37_Rv for each sigma factor (primer sequences used for PCR amplification are listed in Supplementary Table 5). Amplified DNA was digested with the appropriate restriction enzymes, and it was cloned into the corresponding sites in the promoter-probe vector pJEM13 to create in-frame fusions with the *E. coli lacZ* reporter gene. Recombinants were selected based on blue–white selection on kanamycin-containing LB-agar plates and verified by nucleotide sequencing. Recombinant constructs in the donor plasmid (pACYCDuet-1) and/or the target plasmid (pJEM13) were introduced into the *E. coli* BL21 (DE3) strain by electroporation. Transformants were selected on LB-agar plates containing the appropriate antibiotic. Selected transformants were grown in liquid media and induced with IPTG for protein overproduction and subsequent analyses.

### Medium-throughput IPTG induction and β-galactosidase assay

*E. coli* BL21 (DE3) transformants carrying the various combinations of donor and target plasmid pairs were grown overnight in LB broth. Five microlitres of stationary seed cultures were transferred into a 96-well microtiter plate containing, per well, 200 μl LB broth supplemented with the appropriate antibiotics. Plates were incubated at 37 °C until the absorbance at 600 nm (A_600_) of the culture reached 0.15–0.25; then 100 μM IPTG was added to each well, as appropriate, to induce sigma factor gene expression. Following incubation overnight, the density of the culture was determined by A_600_. Cells were collected by centrifugation and resuspended in 200 μl per well of Z buffer (60 mM Na_2_HPO_4_, 40 mM NaH_2_PO_4_ H_2_O, 10 mM KCl, 1 mM MgSO_4_, 50 mM β-mercaptoethanol). Cells were permeabilized by adding 20 μl of freshly prepared 0.05% sodium dodecyl sulfate (SDS) and 20 μl of chloroform (Sigma-Aldrich) to each well, followed by multiple pipetting of the cell suspension with a multi-channel pipettor. Chloroform was allowed to settle to the bottom of the wells, and 25 μl of the aqueous phase were transferred to a fresh microtiter plate for the β-galactosidase assay^57^. The enzymatic reaction was started by adding o-nitrophenyl-β-D-galactopyranoside (5 mM final concentration) and stopped by addition of 75 μl of 1.5 M sodium carbonate after 5 and 10 min in all experiments. Colour intensity was measured at OD420 nm in a Spectramax microplate reader (Molecular Devices Cooperation, Sunnyvale, CA). β-galactosidase activity was calculated as Miller units (MU) by using the formula: MU=1,000 × OD_420_(Time (min) × volume of lysate (ml) × OD_600_)^−1^.

### Detection of sigma factor induction

*E. coli* BL21 (DE3) carrying the pACYCDuet-1 plasmids expressing *M. tuberculosis* sigma factors were grown in LB broth at 37 °C to mid-log phase, and 100 μM IPTG (final concentration) was added. After 2 h, 1-ml culture aliquots were collected and resuspended in 1 × Laemmli buffer (http://cshprotocols.cshlp.org), boiled for 10 min and analysed by 10% SDS–polyacrylamide gel electrophoresis (SDS–PAGE). Perfect Protein^TM^ western blot marker (EMDBiosciences, Madison, WI) was used as molecular weight marker. After SDS–PAGE, protein was transferred to polyvinylidene difluoride membranes for western blot analysis by standard methods (http://cshprotocols.cshlp.org/). Membranes were probed with anti-S tag monoclonal antibody (1:20,000 dilution; EMD Biosciences), followed by 2-h incubation with horseradish peroxidase-conjugated goat anti-mouse-IgG as secondary antibody (1:10,000 dilution; EMD Biosciences). Detection was conducted by chemiluminescence using a luminol-based reagent (20 × LumiGLO Reagent and 20 × Peroxide, Cell Signaling, Boston, MA).

### Construction of anhydrotetracycline-inducible sigma factors

Constructs were generated by Gateway recombination cloning technology (http://www.lifetechnologies.com/us/en/home/life-science/cloning/gateway-cloning/protocols.html), as described^58^. The *sigB* gene was amplified from *M. tuberculosis* genomic DNA by PCR using the primers clo-sigB-attB2 and clo-sigB-attB3 (Supplementary Table 6). These primers introduced the *attB* sites required to clone the PCR product by BP (attBxattP) recombination and a synthetic translational initiation site seven nucleotides upstream of the open reading frame of *sigB*. This *sigB* fragment was then combined by LR (attLxattR) recombination with a codon-usage-adapted TetR and a TetR-controlled promoter, as per standard methods^58^. The resulting plasmid, pGMEH-10M1-*sigB*, allowed for anhydrotetracycline-inducible expression of *sigB* in mycobacteria. LR and BP recombination was performed using clonases from Clontech Laboratories Inc. (Mountainview, CA) according to the manufacturer's instructions. The resulting plasmid constructs were verified by restriction endonuclease mapping and DNA sequencing.

### Treatment of *M. tuberculosis* cultures

*M. tuberculosis* H_37_Rv cultures were grown at 37 °C in 7H9 broth to mid-log phase. For SDS-mediated stress, cultures were treated with a bacteriostatic concentration of SDS (0.03%) for 60 min. For gene-induction experiments with anhydrotetracycline (ATC), 6-ml culture aliquots of *M. tuberculosis* H_37_Rv containing tetracycline-inducible constructs were treated with 1.6 μg ml^−1^ ATC and incubated for an additional 24 h. ATC stocks and ATC-treated cultures were maintained in the dark due to light sensitivity of this compound. At the end of each treatment, 2-ml culture aliquots were collected by centrifugation, and cell pellets were stored for subsequent RNA extraction.

### Quantitative real-time PCR

Bacterial cell pellets were resuspended in 1 ml TRI reagent (Molecular Research Center, Cincinnati, OH) and 0.5 ml zirconia beads (0.1-mm diameter, BioSpec Products, Inc., OH). Cells were disrupted in a bead beater (BioSpec Products, Inc., Bartlesville, OK) by three 45-s pulses, each separated by 10 min incubation on ice. Cells were lysed by adding 100 μl BCP Reagent (Molecular Research Center) and vigorous mixing for 10 min. After another 10 min at room temperature, tubes were centrifuged for 30 min at 12,000*g* at 4 °C. The aqueous phase was transferred to fresh tubes containing 500 μl isopropanol for overnight precipitation. After three cycles of overnight precipitation with isopropanol, the samples were washed with 75% ethanol, air dried and resuspended in diethyl pyrocarbonate-treated H_2_O for storage at −80 °C. Reverse transcription was performed with random hexameric primers and ThermoScript Reverse Transcriptase (Invitrogen). Enumeration of mRNAs was carried out by qPCR using gene-specific primers, molecular beacons and AmpliTaq Gold polymerase (Applied Biosystems, Foster City, CA) in a Stratagene Mx4000 thermal cycler (Agilent Technologies, La Jolla, CA). In the ATC-induced *sigB* expression experiment, ATC-regulated and native copies of *sigB* were distinguished by using copy-specific forward primers for PCR. Nucleotide sequences of PCR primers and molecular beacons are listed in Supplementary Table 7. *M. tuberculosis* 16S rRNA copy number was used as normalization factor to express data as bacterial transcripts per cell.

### Network reconstruction

#### Experimental reconstruction of the sigma factor network

The following procedure was carried out for the β-galactosidase assay results for each target promoter: analysis of variance (ANOVA) was used to test the variance of the readouts for donor sigma factors and controls. All ANOVA tests have rejected the hypothesis of equality of the means of the distributions at *P*<0.01. Therefore, we used Dunnett's *post hoc* test to compare the readouts for each donor sigma factor with the control. A link was created in the network when the mean of the distribution for the donor sigma factor differed significantly from that of the control (*P*<0.05). All statistical analyses were conducted in R.

#### Construction of transcription factor network (without sigma factors)

Direct interactions constituting the transcription factor network were obtained from available ChIP-seq data sets^17^. The following constraints were used to determine the final set of nodes and edges in the network: (i) both regulator and target should be known transcription factors and (ii) transcription factors should have interactions both as regulators and targets.

#### Construction of combined transcription factor–sigma factor regulatory network

Direct regulatory interactions between transcription factors and sigma factors, and between anti-sigma and anti-anti-sigma factors were obtained from our previous *M. tuberculosis* network reconstruction work^59^,^60^, and from ChIP-seq data^17^,^61^.

### Statistical analyses of network patterns

#### Three-node network motifs

MATLAB scripts were used to detect and count the number of occurrences of a given regulatory pattern. The network was then randomized by permuting the sigma factor–transcription factor links. The number of times each given network motif occurred in this randomized network was calculated. Network randomization was repeated 1,000 times, and results were used to calculate mean and standard deviation of the number of occurrences expected by chance. *z*-scores were calculated as [observed−mean(expected)]/std(expected); *z*-scores exceeding 2 were considered significant.

#### Autoregulation

To construct randomized networks to be compared with the sigma factor network, the 41 links of the sigma factor regulatory network were randomly re-assigned between sigma factors 100,000 times. The number of autoregulatory links was calculated for these randomized networks and compared with the sigma factor network in *M. tuberculosis*. The *p* value was estimated by counting how many randomized networks had at least 10 autoregulatory links (the same number as the ‘real' sigma factor network) out of 100,000. The procedure was repeated multiple times. For the comparison between the sigma factor and transcription factor networks in *M. tuberculosis*^17^, the number of autoregulatory nodes was calculated as percentage of the total number of nodes.

### Network topological properties

#### Hierarchy

The hierarchy score maximization algorithm^30^ was used to calculate the hierarchical organization of the sigma factor network. The method runs the algorithm for different number of levels *k* (2–6), and, for each *k*, yields probabilities for each node's assignment to each of the *k* levels. To determine the optimal choice of *k*, the following two measures were calculated for each *k*: (i) the reported corrected hierarchy score that quantifies the enrichment in the downward flow direction relative to expectation^30^, and (ii) the node ambiguity score calculated as the difference between highest and second-highest probabilities assigned for each node (Supplementary Fig. 3). These two measures indicated that *k*=3 was optimal, which was thus considered to be the appropriate number of hierarchical levels best describing the sigma factor network. Since the algorithm is not deterministic, it was run 100 times, and the above calculations were performed on the median probabilities of each node across the 100 runs. These median probabilities were then used to assign each sigma factor to one of the three levels (top, middle and bottom). The hierarchically organized sigma factor network was visualized using Cytoscape (v3.2.1).

To estimate the local hierarchy of the sigma factor network, the in- and out-degrees were calculated for each node, with out-degree (O) being the number of links originating from a node, and in-degree (I) being the number of links terminating at a node. The ratio between the overall connectivity (sum of in- and out-degree for each node) and the ‘hierarchy height', an indicator for the hierarchical rank of each node according to the direction of information flow (the difference between out- and in-degree (O–I) for each node) was calculated for each node^31^. A positive hierarchy height indicates that the information tends to flow away from the node, while a negative hierarchy height implies information flow towards a node. The normalized hierarchy height, NHH=(O−I)/(O+I) defines the three hierarchical levels in the network and membership of the participating nodes.

#### Average path length and clustering coefficient

The igraph package for R (http://igraph.org) was used to calculate average path length and clustering coefficients. To robustly compare the sigma factor and the transcription factor networks, these properties were calculated on random sub-networks containing two-thirds of the total number of nodes and their incident edges in the original networks. The process was repeated 100 times. The resulting distributions of average path lengths and clustering coefficients for the two networks were compared using Wilcoxon's rank-sum test. These network properties were also calculated for ‘deletion mutant' versions of the sigma factor network that were obtained by removing one factor (and all its incident edges) at a time from the network.

#### Out- and in-degree distributions of sub-networks

The in- and out-degree distributions for the sigma factor and transcription factor networks were calculated using the igraph package. Since the transcription factor network (67-nodes, 198-edges) is larger than the sigma factor network (13-nodes, 41-edges), a comparable in- and out-degree distribution was obtained for the transcription factor network as the median of 100 randomly selected 13-node subgraphs (13 nodes along with their incident incoming and outgoing edges) from the original network.

#### Community detection algorithms

Communities of sigma factors were identified in the network by first representing the directed network as an undirected bipartite network where (i) each sigma factor was represented by two nodes, a protein node and a gene node, and (ii) a direct link between two nodes x and y that could be of two possible types: either from protein node x to gene node y (‘protein x regulates gene y'), or from gene node x to protein node x (‘gene x encodes and therefore by definition regulates protein x'). This method is preferred to those disregarding link directionality and therefore yielding a unipartite network because it preserves the maximal amount of information for the analysis. An algorithm^62^ that combines spectral bisectioning^63^ with a variant of Kernighan–Lin-type refinement^64^ and agglomeration was then used to find a node partition that maximizes Barber's bipartite modularity^65^. This method identifies ‘biclusters' consisting of both genes and proteins that are highly connected compared with what would be expected if the bipartite links were randomly placed. The community detection algorithm is partially stochastic; by repeatedly running it (10,000 times), an ensemble of partitions with similar modularities can be identified. The full ensemble was then analysed to determine *p*_*ij*_ the probability that each pair of nodes is biclustered together^66^. The results of such analyses were visualized in a ‘heat-map' correlation matrix plot. To better visualize the results in this plot, the order of the nodes was optimized using simulated annealing^67^ with a cost function of 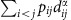, where 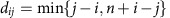 is a measure of distance from the diagonal of the *i*th, *j*th block of the matrix assuming periodic boundary conditions on the node ordering, with *α*=1 here^34^. Periodic boundary conditions were used to avoid biasing the position of particular nodes.

### Consensus binding motif search

#### sigE promoter region

The MEME suite was used for motif discovery (MEME^68^) and motif scanning (MAST^69^). Two types of query sequences were used: (i) published consensus binding motifs for SigA, SigE and SigH (Supplementary Table 2), and (ii) MEME-generated consensus motifs from published target sequences for SigA (12 targets^70^), SigE (9 targets^21^) and SigH (7 targets^22^). The upstream region of *sigE* (the ∼500-bp sequence used for the *E. coli lacZ* fusion) was used for motif scanning by MAST.

#### Promoter regions of all sigma factors

All published consensus binding motifs for sigma factors (Supplementary Table 2) were used to scan ∼500-bp regions upstream of all 13 sigma factors by MAST.

### Mining reported direct sigma–sigma interactions

Known direct sigma–sigma interactions were obtained from ChIP data and *in vitro* transcription assays (Supplementary Table 1). Transcription factor overexpression ChIP-seq data specific to sigma–sigma interactions were obtained from ref. 17, the MTB Network Portal (networks.systemsbiology.net/mtb/) and ref. 51. ChIP-on-chip data were obtained from ref. 16. *In vitro* transcription assay data were obtained with PubMed queries for ‘*in vitro* transcription assay' followed by sigma factor gene name or corresponding Rv number. Transcription factor overexpression microarray data for sigma–sigma interactions were obtained from refs 71, 72. A threshold of *P*<0.01 was applied for the selection of ChIP-seq and microarray data.

## Additional information

**How to cite this article:** Chauhan, R. *et al*. Reconstruction and topological characterization of the sigma factor regulatory network of *Mycobacterium tuberculosis*. *Nat. Commun.* 7:11062 doi: 10.1038/ncomms11062 (2016).

## Supplementary Material

Supplementary Information Supplementary Figures 1-6, Supplementary Tables 1-7 and Supplementary References
