# Supplementary material for: Reconstruction and topological characterization of the sigma factor regulatory network of Mycobacterium tuberculosis
Source: Nat Commun. 2016 Mar 31;7:11062. doi: 10.1038/ncomms11062 (PMC4821874; doi:10.1038/ncomms11062)
Supplement: Supplementary Information — Supplementary Figures 1-6, Supplementary Tables 1-7 and Supplementary References [file ncomms11062-s1.pdf]

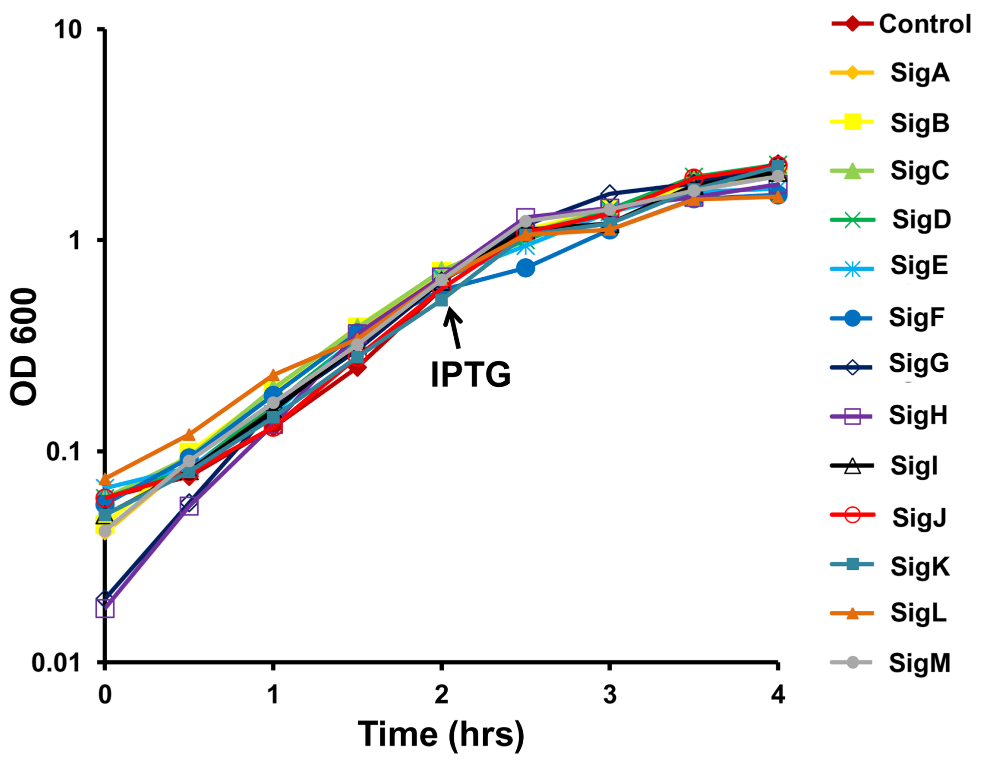

Supplementary Figure 1. Growth curves of IPTG-induced cultures of BL21(DE3) *E. coli* strains overexpressing sigma factor proteins of *M. tuberculosis*.

E. coli BL21 (DE3) carrying empty pACYCDuet-1 expression vector (control) and vectors overexpressing each of 13 sigma factors of *M. tuberculosis* were grown to cell density of $A_{600} = 0.5$, and treated with 100 μm IPTG (arrow) to induce sigma factor protein expression. Cell density of the cultures was recorded every 30 minutes for two hours after induction.

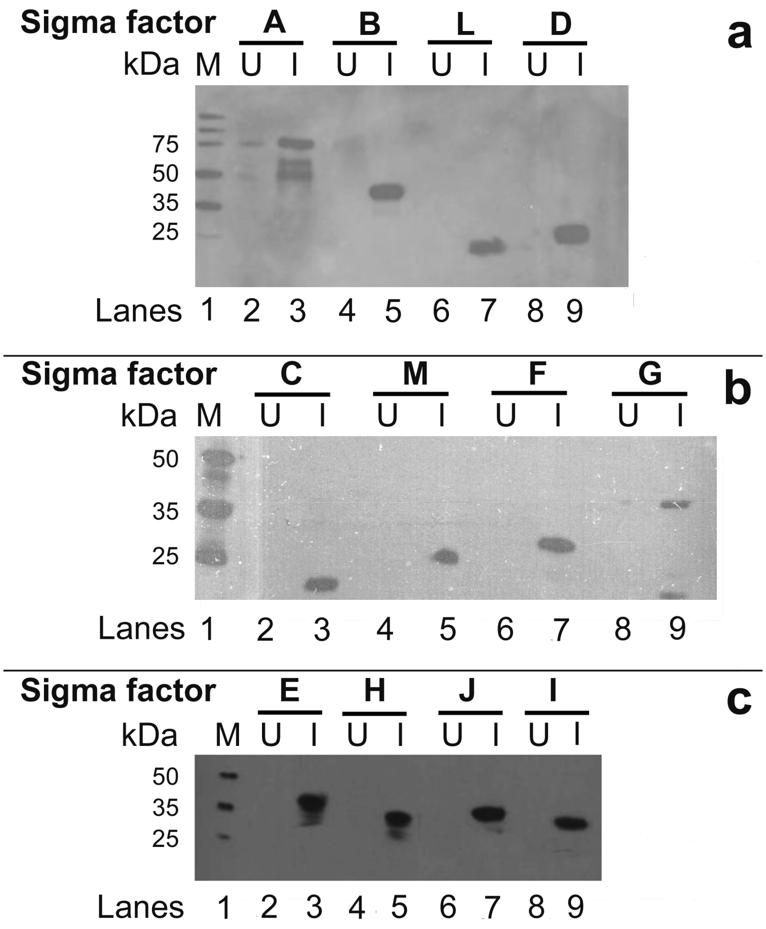

Supplementary Figure 2. Western blot analysis of BL21(DE3) *E. coli* strains overexpressing the sigma factor proteins.
E. coli BL21(DE3) carrying the pACYCDuet-1 plasmids expressing *M. tuberculosis sigA* through *sigM* were grown in LB broth at 37°C to mid-log phase, and 100 M IPTG was added. After two hours, 1-ml culture aliquots were harvested, resuspended in electrophoresis sample buffer, and boiled for 10 min. Protein was separated by 10 % SDS-PAGE, transferred to PVDF membranes, and probed with anti-S tag antibody. Detection was with two-hr incubation with horseradish peroxidase-conjugated goat anti-mouse-IgG as secondary antibody. The proteins were detected by chemiluminescence using Western Blotting Luminol Reagent. All sigma factors were detected by western blot analysis, except SigK. However, the *E. coli* clone expressed SigK activity, since we found two SigK targets in the *E. coli* assay (Fig. 2), which were valid in *M. tuberculosis* (Fig. 4d and PMID 17064366). Each panel (a–c) shows results for four sigma factors, as indicated at the top of each lane pair: U = uninduced sample; I = IPTG-induced sample. M = Marker, MW = Molecular weight.

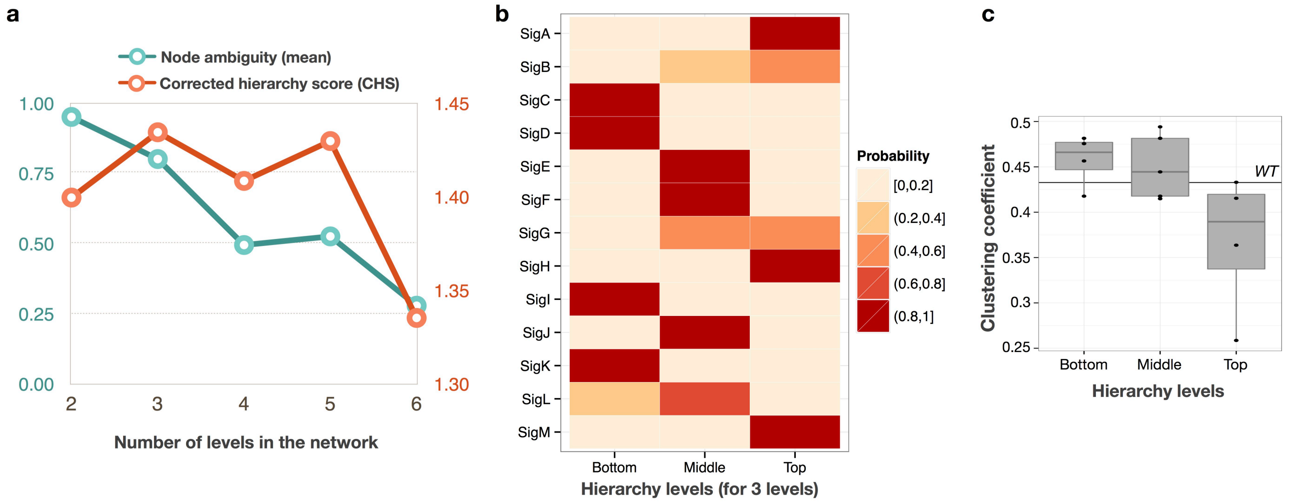

Supplementary Figure 3. Hierarchy in the sigma factor network of *M. tuberculosis*.
(**a**) Measures of network hierarchy for the sigma factor network for varying number of levels from 2 to 6: node ambiguity (blue) equals the mean difference between the highest and the penultimate probabilities across levels for each node; corrected hierarchy score (orange) quantifies the enrichment in downward flow relative to expectation for a given hierarchical organization [1]. High node ambiguity indicates an unambiguous assignment of nodes to levels, and high hierarchy score indicates optimal hierarchical organization. (**b**) The heatmap shows the probability that each node (row) belongs to one of the three levels of the hierarchy (Top, Middle and Bottom, corresponding to 'Number of levels' equal to 3 in panel **a**). (**c**) Association of node hierarchy with clustering coefficient. The clustering coefficients of the sigma factor network were calculated after removing individual nodes in a given hierarchical level, and the resulting coefficients for nodes in each of the three hiearchical levels were plotted as boxplots, with the points representing individual values. The horizontal line corresponds to the clustering coefficient of the wild type (WT) network.

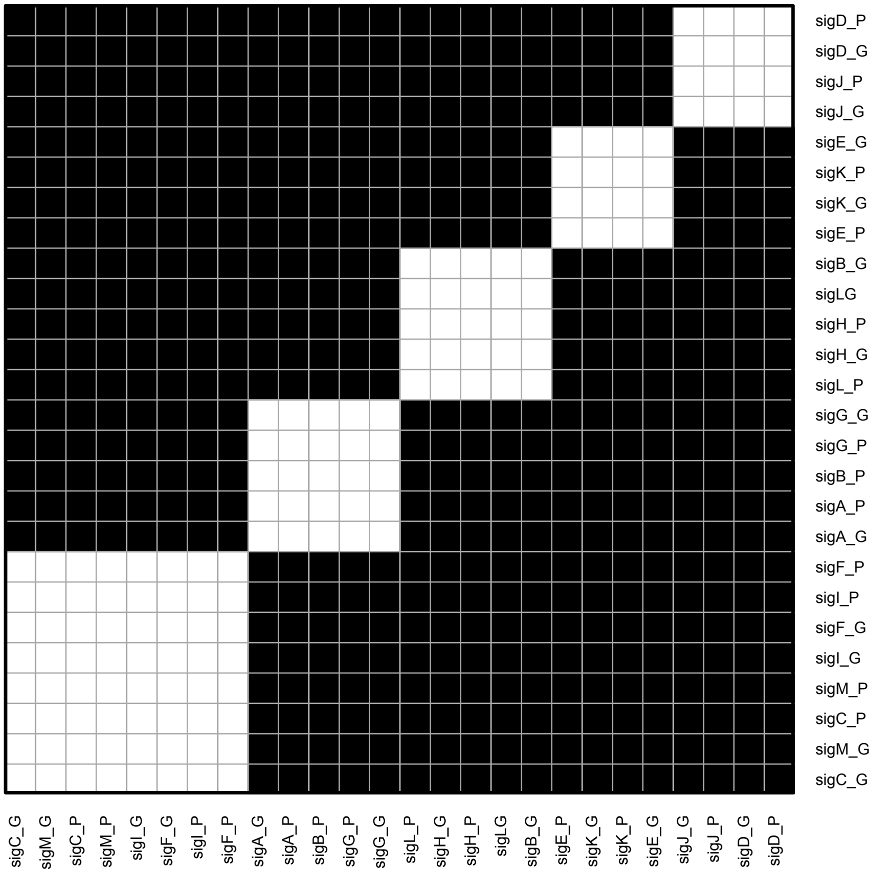

Supplementary Figure 4. Correlation matrix of community membership.
A heatmap of the correlation matrix showing the results of the community analysis from an ensemble of 10,000 community detection runs performed on the sigma factor network in Fig. 3. Each sigma factor (for example, sigA) is represented with a gene node (for example, sigA_G) and a protein node (for example, sigA_P). The matrix elements show the probability that each pair of nodes is found to be in the same community. White indicates the case when the two nodes (along the row and column) were always found together in the same community; black indicates the case when they were never found together. The five-community partition shown in the heatmap has a modularity of 0.46. The average modularity for random networks is 0.33, with a variance of 1.4E-3. The resulting *z*-score of the sigma factor network is 96.24, indicating significantly high modularity (i.e., community structure) of the network.

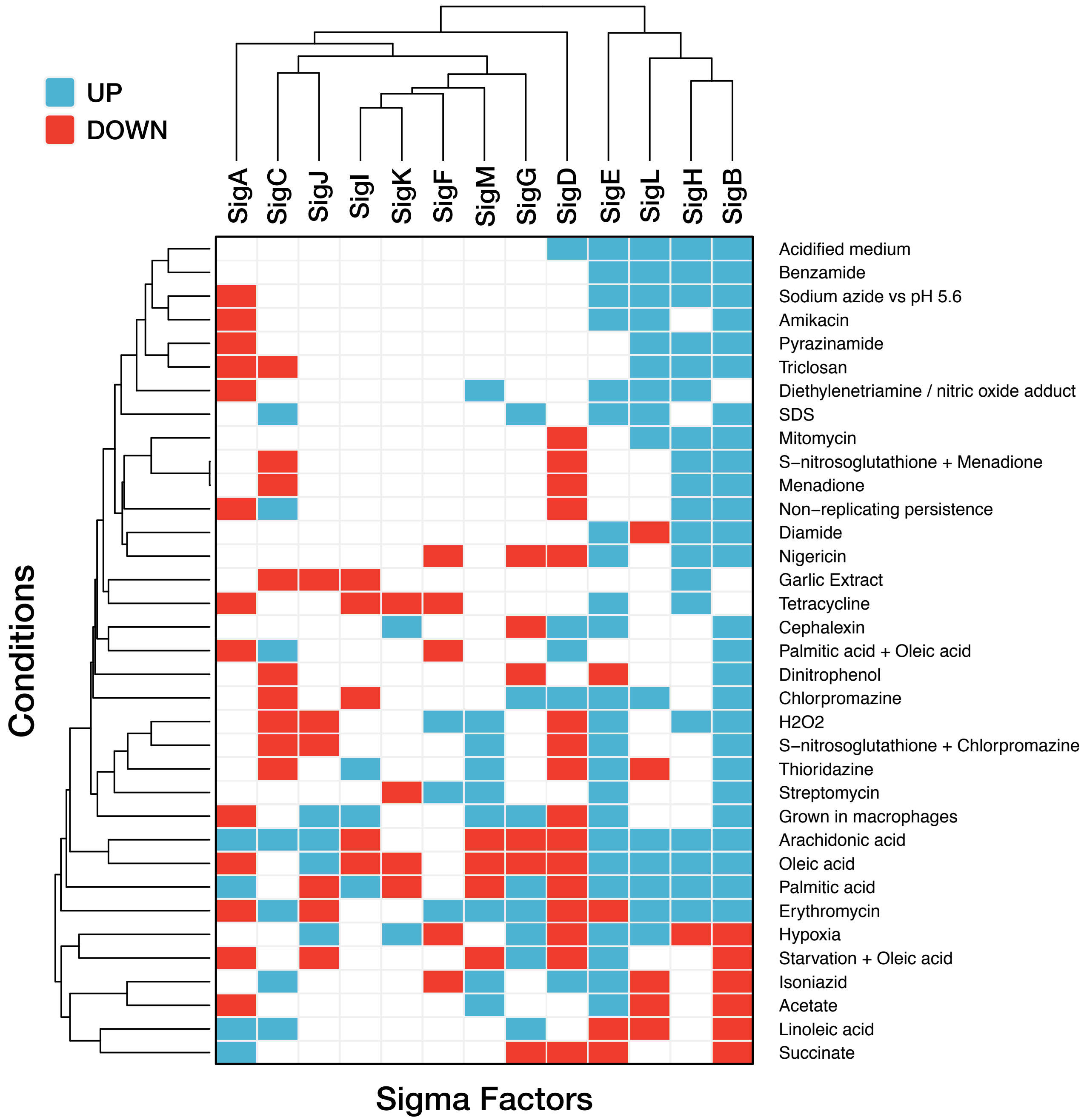

Supplementary Figure 5. Coexpression analysis of sigma factors across conditions. Expression data were obtained from a total of 40 genome-wide data sets listed in the TB DataBase (www.tbdb.org). For the present analysis, 103 conditions (>800 experiments) were selected in which at least one sigma factor was differentially expressed, with significance absolute *z*-score ≥2. When treatment conditions were similar (for example, treatment with a particular stressor at multiple concentrations and/or for different time lengths), they were combined; for each group of similar conditions, the most significant expression change (highest absolute *z*-score) for each sigma factor was taken. The selected values were abstracted for each sigma factor into +1 (up-regulation) and -1 (down-regulation). Absence of significant differential expression was converted into zero. The

resulting heatmap shown in the figure represents hierarchical clustering (using Euclidean distance) of differential expression of sigma factors (columns) vs. experimental conditions (rows). Blue and red indicate up- and down-regulation, respectively; white indicates no significant expression changes. Only conditions in which ≥4 sigma factors were differentially regulated are shown (clustering results did not change when the list of conditions included also those with ≥3 differentially regulated sigma factors). *sigB*, *sigE*, *sigH*, and *sigL* formed a distinct cluster, in agreement with reciprocal regulation among these factors [ELH –> B; BH –> L; L –> H; H –> E (Figure 2 and Supplementary Table 1)]. In addition, *sigC*, *sigF*, *sigI,* and *sigM*, which constitute the most robust community within the network, clustered together, along with *sigJ* and *sigK*, which are intermediate nodes bridging the four-node community and the larger network.

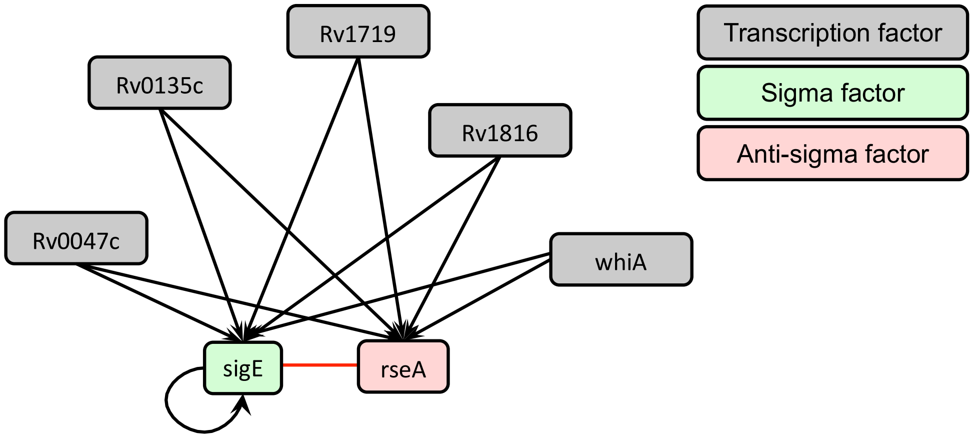

Supplementary Figure 6. An example of the network structure where a transcription factor regulates a sigma factor and its cognate anti-sigma factor.
Transcription factors that regulate *sigE* and *rseA* (cognate anti-sigma). Since *sigE* and *rsaE* are expressed from separate promoters, their joint regulation is not a consequence of bicistronic expression.

Supplementary Table 1. Previously reported direct sigma-sigma interactions

Gene name	Rv number	ChIP data	*In vitro* transcription assays
**SigA**	**Rv2703**	SigA[a]	—
**SigB**	**Rv2710**	—	SigB ref.[2] but not ref.[3]
**SigC**	**Rv2069**	—	—
**SigD**	**Rv3414c**	SigD[a,b,c]	SigD ref.[4]
**SigE**	**Rv1221**	—	SigB[d] ref.[3,5]
**SigF**	**Rv3286c**	SigF[a]	SigB ref.[3]<br>SigC ref.[2]<br>SigF[d] ref.[6]
**SigG**	**Rv0182c**	—	—
**SigH**	**Rv3223c**	SigE[a], SigH[a,b,c]	SigB ref.[3,7]<br>SigE[d] ref.[5]
**SigI**	**Rv1189**	—	—
**SigJ**	**Rv3328c**	—	SigI[d] ref.[8]
**SigK**	**Rv0445c**	—	—
**SigL**	**Rv0735**	SigE[a], SigL[c]	SigB ref.[3]<br>SigL ref.[9]
**SigM**	**Rv3911**	—	SigM ref.[10]

[a] Transcription Factor Over-Expression (TFOE) ChIP-Seq data [11]
[b] TFOE ChIP-Seq data, Mtb network portal [12]
[c] ChIP on chip data [13]
[d] Links that are also revealed by TFOE Microarray data [14,15].
'—' indicates 'not done' or 'not significant'

Supplementary Table 2. Published consensus binding motifs

Sigma factor	Promoter consensus sequence -35 -10	References	Previously reported targets[a]
SigA	TTGCGA – [N18] – TANNNT	ref.[16]	sigA
SigB	NGTGG – [N14-18] – NNGNNG	ref.[2]	sigB
SigC	SSSAAT – [N16-20] – CGTSSS	ref.[17]	—
SigD	GTAACGct – – – AT-rich region<br>AGAAAG – [N16-20] – CGTTAA	refs.[4,18]	sigD
SigE	gGGAACYa – [N15-16] – cGTT	ref.[19]	sigB[b]
SigF	GGWWT – [N16-17] – GGGTAY	ref.[13]	sigB, sigC, sigF
SigG	GCGNGT – [N15-18] – CGANCA	ref.[20]	—
SigH	gGGAAYA – [N16-17] – cGTT	ref.[5]	sigB[b], sigE[b], sigH
SigI	unknown	—	—
SigJ	GTCACA – [N16] – CGTCCT	ref.[8]	sigI[b]
SigK	CCATCC – [N15] – CCGAAT	ref.[21]	—
SigL	TGAACC – [N16] – CGTgtc	ref.[9]	sigB, sigE, sigL[b]
SigM	GGAAC – [N16-18] – CGTCR<br>GGGAACC – [N17] – gtCcgA	refs.[22,10]	sigM[b]

[a] List of direct sigma factor targets as in Supplementary Table 1.
[b] Sigma factor targets identified by MAST using the consensus motifs in the second column.
No consensus binding motif was found for any of the 11 sigma factors analyzed by ChIP-Seq [11].

Supplementary Table 3. Known anti-sigma factors

Anti-sigma (rv no.)	Cognate sigma	Sigma-anti-sigma coregulation	References
**RsdA (rv3413c)**	SigD	likely (6 bp intergenic)	refs.[23,24]
**RseA (rv1222)**	SigE	no	refs.[25,26]
**UsfX (rv3287c)**	SigF*	yes	refs.[6,27,28]
**RshA (rv3221A)**	SigH	yes	refs.[29-31]
**RskA (rv0444c)**	SigK	yes	refs.[32,33]
**RslA (rv0736)**	SigL	yes	refs.[3,9,34]
**RsmA (rv3912)**	SigM	likely (16 bp intergenic)	ref.[35]

bp = base pairs
* Anti-anti-sigma factors are known only for SigF: RsfA (rv1365c), RsfB (rv3687c) (PMID: 12354223).

Supplementary Table 4. Primers used for expression from donor plasmid (pACYCDuet-1)

Primer code	Primer sequence (5'—3')
SigA_fwd	AAATTAGATCTTGTGGCAGCGACCAAAGCAAG
SigA_rev	AAATTGGTACCGTCCAGGTAGTCGCGCAGGA
SigB_fwd	AAATTAGATCTTATGGCCGATGCACCCACAAG
SigB_rev	AAATTGGTACCGCTGGCGTACGACCGCAGCC
SigC_fwd	AAATTAGATCTTATGACCGCGACGGCAGCGA
SigC_rev	AAATTGGTACCGCCGGTGAGGTCGTCGGGCT
SigD_fwd	AAATTGGATCCTATGGTCGATCCGGGAGTTAG
SigD_rev	AAATTGGTACCCGCATAGTCACCTGCCGCAA
SigE_fwd	AAATTGGATCCTATGGAACTCCTCGGCGGACC
SigE_rev	AAATTGGTACCGCGAACTGGGTTGACGTGAA
SigF_fwd	AAATTAGATCTTGTGACGGCGCGCGCTGCCGG
SigF_rev	TTCTCCAACTGATCCCGTAGCC
SigG_fwd	AAATTAGATCTTATGCGCACATCGCCGATGCC
SigG_rev	AAATTCTCGAGCAGCGAATCGGGCAGGCCGA
SigH_fwd	AAATTAGATCTTATGGCCGACATCGATGGTGT
SigH_rev	AAATTCTCGAGTGACGACACCCCCTCGTGCG
SigI_fwd	AAATTCAATTGTATGTCGCAACACGACCCGGT
SigI_rev	AAATTGGTACCACCGCCGCCGAGTTCGGCCC
SigJ_fwd	AAATTAGATCTTATGGAGGTTTCCGAATTCGA
SigJ_rev	AAATTGGTACCATTCCGGTGATGCCTGCCGC
SigK_fwd	AAATTGGATCCTATGACCGGACCGCCACGGCT
SigK_rev	AAATTGGTACCTGACACGTCCAGGCAGTTGC
SigL_fwd	AAATTAGATCTTGTGGCTCGTGTGTCGGGCGC
SigL_rev	AAATTGGTACCTCGAGTAACTCCCAGTTCCT
SigM_fwd	AAATTAGATCTTATGCCGCCACCGATTGGTTA
SigM_rev	AAATTGGTACCTGCCCGGTGGCAATAGCCAG

Supplementary Table 5. Primers used for expression from target plasmid (pJEM13)

Primer code	Primer sequence (5'—3')
SigB_fwd1	ATTGGATCCCCGTCTGTTGGCCGGCGTTC
SigB_rev1	ATTGGTACCGCTGTCAACCCGGCTTGTGG
SigC_fwd1	ATTGGATCCTTGGGCCACCGGGGAGATCG
SigC_rev1	ATTGGTACCGGCGAGTGCGGTAACGGCCT
SigD_fwd1	ATTGGATCCTCCCCGCTCGTGGCGGACCG
SigD_rev1	ATTGGTACCGTCTCCTGCCACGGCCTCCG
SigE_fwd1	ATTGGATCCCACCGCCGGTGTTACCGCCC
SigE_rev1	ATTGGTACCATGAGACATGCTGGTCGGAC
SigF_fwd1	ATTGGATCCACCTATCGTGACCCCGTCGA
SigF_rev1	ATTGGTACCGACACCGCGTTGGCGCCCCT
SigG_fwd1	ATTGGATCCGGTCGGTGTTGTAAGCCTGG
SigG_rev1	ATTGGTACCCACCCGGACTGAACGGAATT
SigH_fwd1	ATTGGATCCGACGATCGGCAGTGCCTGGC
SigH_rev1	ATTGGTACCAGGCTGCAGACCCGCCGAAC
SigI_fwd1	ATTAGTACTCCGTGGGCGCCCGAGTCCGG
SigI_rev1	ATTGGATCCTTCCGATGCGCCCGCCAGGCCG
SigJ_fwd1	ATTGGATCCGAACAATGGCAGGCCGGTGA
SigJ_rev1	ATTGGTACCCGACATGAGATGCTGTCGCA
SigK_fwd1	ATTGGATCCGTCGGTATCACCTTCGAAGC
SigK_rev1	TTGGTACCCAACAGGGCGTCCAGGTCGC
SigL_fwd1	ATTGCGCCCTGAGCGACGCCGAC
SigL_rev1	ATTCATCAACGCGGCTTCAGCGG
SigM_fwd1	ATTGGATCCTTCGGCGCCGCCCGGCAGCG
SigM_rev1	ATTGGTACCGCCCCCGAAACCCACGGCCG
SigEP1_fwd	AAATTGGATCCATTGCTCATATATGGCCCAT
SigEP2_fwd	AAATTGGATCCCCTCGGCGGACCCCGGGTTG

Supplementary Table 6. Primers used for expression from ATC-inducible plasmid (pGMEH-10M1)

Primer code	Primer sequence (5'—3')
**clo-sigB-attB2**	GGGGACAGCTTTCTTGTACAAAGTGGAAGGAGGTATACATATGGCCGATGCACCCACAAG
**clo-sigB-attB3**	GGGGACAACTTTGTATAATAAAGTTGGGCTCAGGATGTCCAGCTTCAG

Supplementary Table 7. List of qPCR primers and molecular beacons (MB)

Gene	qPCR primers (5'-3')
ideR	Fwd: AACGCACGAGTAACCGTCGAAAC<br>Rev: TCAGACTTTCTCGACCTTGACCGC<br>MB: CCCCGGCGGCGGCGTGACCATCGTCATCCCGGGG
mpt70	Fwd: CTCGAACAATCCGGAGTTGACA<br>Rev: AACAGCGGTCAGTACACGGTGT<br>MB: GCAGCCAGCTCAATCCGCAAGTAAACCTGGGCTGC
sigD	Fwd: ATTCCTGGCGTTTCTGTACGGCAT<br>Rev: TCTCAAGCAATTCGTTCATCCGGG<br>MB: CGCGTGTATCCCGCCGAAACGCTTCCTGCACGCG
sigK	Fwd: AATTCTACGACCACACCAAGTCGC<br>Rev: GTTCCGCCACACCTCAAGATAGAT<br>MB: ACGGGGGTATGGACTGGTGATGCGGGTCCCCGT
sigG	Fwd: AAATTCCGTTCAGTCCGGGTGGTA<br>Rev: AGCACACTCACGTCAATGAGCCTA<br>MB: ACCCGCCGGTGAGAGTGTCGGAGACTCGCGGGT
sigL	Fwd: GAATTGCCGAAGGAACGGTGAAGTC<br>Rev: AACTCCCAGTTCCTGCAGAGTGA<br>MB: ACGGCGTGAAGTCGCGATTGCACTACGCCCGCCGT
sigF	Fwd: TCTTGACCAGATCGAGAATCGGGA<br>Rev: AGTCGAAGAACCTGAGCACCAAGA<br>MB: CGCGTGGAACGGTCTTGGTGCTCAGGTCACGCG
sigC	Fwd: GCGTTTATCAAAGCCACCCAGCAA<br>Rev: ATCGCTCGTAGGAATGTCTCTTGG<br>MB: ACCCGCCAAAGCCACCCAGCAAGACGTGTGCGGGT
sigH	Fwd: TGTCGGTCGCGGCCTCTACAT<br>Rev: GCTTCTCACCCAACAGGCTCGTC<br>MB: AGGGCCGAGGGGAGTCTGGTGTCAACGGGCCCT
16s rRNA	Fwd: ATGACGGCCTTCGGGTTGTAA<br>Rev: CGGCTGCTGGCACGTAGTTG<br>MB: CCCCGCCGACGAAGGTCCGGGTTCTCGCGGGG
lacZ	Fwd: TTGTTGCCATTGCTACAGGCATCG<br>Rev: TGTAACTCGCCTTGATCGTTGGGA<br>MB: ACCCGGGGTGTCACGCTCGTCGTTTGGCCGGGT

Supplementary References

1 Cheng, C. *et al.* An approach for determining and measuring network hierarchy applied to comparing the phosphorylome and the regulome. *Genome Biol* **16**, 63, doi:10.1186/s13059-015-0624-2 (2015).

2 Lee, J. H., Karakousis, P. C. & Bishai, W. R. Roles of SigB and SigF in the Mycobacterium tuberculosis sigma factor network. *J Bacteriol* **190**, 699-707 (2008).

3 Dainese, E. *et al.* Posttranslational regulation of Mycobacterium tuberculosis extracytoplasmic-function sigma factor sigma L and roles in virulence and in global regulation of gene expression. *Infect Immun* **74**, 2457-2461 (2006).

4 Raman, S., Hazra, R., Dascher, C. C. & Husson, R. N. Transcription regulation by the Mycobacterium tuberculosis alternative sigma factor SigD and its role in virulence. *J Bacteriol* **186**, 6605-6616, doi:10.1128/JB.186.19.6605-6616.2004 (2004).

5 Raman, S. *et al.* The alternative sigma factor SigH regulates major components of oxidative and heat stress responses in Mycobacterium tuberculosis. *J Bacteriol* **183**, 6119-6125 (2001).

6 Beaucher, J. *et al.* Novel Mycobacterium tuberculosis anti-sigma factor antagonists control sigmaF activity by distinct mechanisms. *Mol Microbiol* **45**, 1527-1540 (2002).

7 Manganelli, R. *et al.* Role of the extracytoplasmic-function sigma factor sigma(H) in Mycobacterium tuberculosis global gene expression. *Mol Microbiol* **45**, 365-374 (2002).

8 Homerova, D., Halgasova, L. & Kormanec, J. Cascade of extracytoplasmic function sigma factors in Mycobacterium tuberculosis: identification of a sigmaJ-dependent promoter upstream of sigI. *FEMS Microbiol Lett* **280**, 120-126, doi:FML1054 [pii] 10.1111/j.1574-6968.2007.01054.x (2008).

9 Hahn, M. Y., Raman, S., Anaya, M. & Husson, R. N. The Mycobacterium tuberculosis extracytoplasmic-function sigma factor SigL regulates polyketide synthases and secreted or membrane proteins and is required for virulence. *J Bacteriol* **187**, 7062-7071, doi:10.1128/JB.187.20.7062-7071.2005 (2005).

10 Raman, S. *et al.* Mycobacterium tuberculosis SigM positively regulates Esx secreted protein and nonribosomal peptide synthetase genes and down regulates virulence-associated surface lipid synthesis. *J Bacteriol* **188**, 8460-8468, doi:10.1128/JB.01212-06 (2006).

11 Minch, K. J. *et al.* The DNA-binding network of Mycobacterium tuberculosis. *Nat Commun* **6**, 5829, doi:10.1038/ncomms6829 (2015).

12 Turkarslan, S. *et al.* A comprehensive map of genome-wide gene regulation in Mycobacterium tuberculosis. *Sci Data* **2**, 150010, doi:10.1038/sdata.2015.10 (2015).

13 Rodrigue, S. *et al.* Identification of mycobacterial sigma factor binding sites by chromatin immunoprecipitation assays. *J Bacteriol* **189**, 1505-1513 (2007).

14 Peterson, E. J. *et al.* A high-resolution network model for global gene regulation in Mycobacterium tuberculosis. *Nucleic Acids Res* **42**, 11291-11303, doi:10.1093/nar/gku777 (2014).

15 Rustad, T. R. *et al.* Mapping and manipulating the Mycobacterium tuberculosis transcriptome using a transcription factor overexpression-derived regulatory network. *Genome Biol* **15**, 502, doi:10.1186/PREACCEPT-1701638048134699 (2014).

16 Agarwal, N. & Tyagi, A. K. Mycobacterial transcriptional signals: requirements for recognition by RNA polymerase and optimal transcriptional activity. *Nucleic Acids Res* **34**, 4245-4257, doi:10.1093/nar/gkl521 (2006).

17 Sun, R. *et al.* Mycobacterium tuberculosis ECF sigma factor sigC is required for lethality in mice and for the conditional expression of a defined gene set. *Mol Microbiol* **52**, 25-38 (2004).

18 Calamita, H. *et al.* The Mycobacterium tuberculosis SigD sigma factor controls the expression of ribosome-associated gene products in stationary phase and is required for full virulence. *Cell Microbiol* **7**, 233-244 (2005).

19 Song, T., Song, S. E., Raman, S., Anaya, M. & Husson, R. N. Critical role of a single position in the -35 element for promoter recognition by Mycobacterium tuberculosis SigE and SigH. *J Bacteriol* **190**, 2227-2230 (2008).

20 Lee, J. H., Geiman, D. E. & Bishai, W. R. Role of stress response sigma factor SigG in Mycobacterium tuberculosis. *J Bacteriol* **190**, 1128-1133, doi:10.1128/JB.00511-07 (2008).

21 Veyrier, F., Said-Salim, B. & Behr, M. A. Evolution of the mycobacterial SigK regulon. *Journal of bacteriology* **190**, 1891-1899, doi:10.1128/JB.01452-07 (2008).

22 Agarwal, N., Woolwine, S. C., Tyagi, S. & Bishai, W. R. Characterization of the Mycobacterium tuberculosis sigma factor SigM by assessment of virulence and identification of SigM-dependent genes. *Infect Immun* **75**, 452-461, doi:10.1128/IAI.01395-06 (2007).

23 Jaiswal, R. K., Prabha, T. S., Manjeera, G. & Gopal, B. Mycobacterium tuberculosis RsdA provides a conformational rationale for selective regulation of sigma-factor activity by proteolysis. *Nucleic Acids Res* **41**, 3414-3423, doi:10.1093/nar/gks1468 (2013).

24 Thakur, K. G., Jaiswal, R. K., Shukla, J. K., Praveena, T. & Gopal, B. Over-expression and purification strategies for recombinant multi-protein oligomers: a case study of Mycobacterium tuberculosis sigma/anti-sigma factor protein complexes. *Protein Expr Purif* **74**, 223-230, doi:10.1016/j.pep.2010.06.018 (2010).

25 Dona, V. *et al.* Evidence of complex transcriptional, translational, and posttranslational regulation of the extracytoplasmic function sigma factor sigmaE in Mycobacterium tuberculosis. *J Bacteriol* **190**, 5963-5971 (2008).

26 Barik, S., Sureka, K., Mukherjee, P., Basu, J. & Kundu, M. RseA, the SigE specific anti-sigma factor of Mycobacterium tuberculosis, is inactivated by phosphorylation-dependent ClpC1P2 proteolysis. *Mol Microbiol* **75**, 592-606, doi:10.1111/j.1365-2958.2009.07008.x (2010).

27 Malik, S. S., Luthra, A., Srivastava, S. K. & Ramachandran, R. Mycobacterium tuberculosis UsfX (Rv3287c) exhibits novel nucleotide binding and hydrolysis properties. *Biochem Biophys Res Commun* **375**, 465-470, doi:10.1016/j.bbrc.2008.08.043 (2008).

28 Malik, S. S., Luthra, A. & Ramachandran, R. Interactions of the M. tuberculosis UsfX with the cognate sigma factor SigF and the anti-anti sigma factor RsfA. *Biochim Biophys Acta* **1794**, 541-553, doi:10.1016/j.bbapap.2008.11.007 (2009).

29 Song, T., Dove, S. L., Lee, K. H. & Husson, R. N. RshA, an anti-sigma factor that regulates the activity of the mycobacterial stress response sigma factor SigH. *Mol Microbiol* **50**, 949-959 (2003).

30 Jeong, E. H. *et al.* RshA mimetic peptides inhibiting the transcription driven by a Mycobacterium tuberculosis sigma factor SigH. *Biochem Biophys Res Commun* **339**, 392-398, doi:10.1016/j.bbrc.2005.11.032 (2006).

31 Kumar, S. *et al.* Interaction of Mycobacterium tuberculosis RshA and SigH is mediated by salt bridges. *PLoS ONE* **7**, e43676, doi:10.1371/journal.pone.0043676 (2012).

32 Said-Salim, B., Mostowy, S., Kristof, A. S. & Behr, M. A. Mutations in Mycobacterium tuberculosis Rv0444c, the gene encoding anti-SigK, explain high level expression of MPB70 and MPB83 in Mycobacterium bovis. *Mol Microbiol* **62**, 1251-1263, doi:10.1111/j.1365-2958.2006.05455.x (2006).

33 Malkhed, V., Gudlur, B., Kondagari, B., Dulapalli, R. & Vuruputuri, U. Study of interactions between Mycobacterium tuberculosis proteins: SigK and anti-SigK. *J Mol Model* **17**, 1109-1119, doi:10.1007/s00894-010-0792-7 (2011).

34 Thakur, K. G., Praveena, T. & Gopal, B. Structural and biochemical bases for the redox sensitivity of Mycobacterium tuberculosis RslA. *J Mol Biol* **397**, 1199-1208, doi:10.1016/j.jmb.2010.02.026 (2010).

35 Sklar, J. G., Makinoshima, H., Schneider, J. S. & Glickman, M. S. M. tuberculosis intramembrane protease Rip1 controls transcription through three anti-sigma factor substrates. *Mol Microbiol* **77**, 605-617, doi:10.1111/j.1365-2958.2010.07232.x (2010).
